# Supplementary material for: Unsupervised Clustering of Missense Variants in HNF1A Using Multidimensional Functional Data Aids Clinical Interpretation
Source: Am J Hum Genet. 2020 Sep 9;107(4):670–82. doi: 10.1016/j.ajhg.2020.08.016 (PMC7536579; doi:10.1016/j.ajhg.2020.08.016)
Supplement: Document S1. Figures S1–S7 and Tables S3–S5 [file mmc1.pdf]

The American Journal of Human Genetics, Volume *107*

Supplemental Data

Unsupervised Clustering of Missense Variants in *HNF1A* Using Multidimensional Functional Data Aids Clinical Interpretation

Sara Althari, Laeya A. Najmi, Amanda J. Bennett, Ingvild Aukrust, Jana K. Rundle, Kevin Colclough, Janne Molnes, Alba Kaci, Sameena Nawaz, Timme van der Lugt, Neelam Hassanali, Anubha Mahajan, Anders Molven, Sian Ellard, Mark I. McCarthy, Lise Bjørkhaug, Pål Rasmus Njølstad, and Anna L. Gloyn

SUPPLEMENTAL DATA

Contents

Figure S1. Ethnic composition of a multi-consortia led exome sequencing study

Figure S2. Division of exome-identified non-synonymous missense alleles

Figure S3. Analytical pipeline for unsupervised stratification of HNF1A missense alleles along the in vivo glycaemic spectrum using multi-dimensional in vitro functional data

Figure S4. Assessment of the optimal number of clusters

Figure S5. Functional studies of protein variants (Bergen)

Figure S6. Functional studies of protein variants (Oxford)

Figure S7. Clustering alignment of *HNF1A* missense variants shared between both centres at Oxford and Bergen

Table S1. Exome-detected *HNF1A* missense alleles

Table S2. MODY reference alleles

Table S3. Scores for tissue specific isoform expression and functional domain

Table S4. Features of *HNF1A* allele carriers documented in UK Registry

Table S5. Features of *HNF1A* allele carriers documented in Norwegian Registry

Table S6. Clinical features discordant *HNF1A* alleles in UK Registry

Table S7. Clinical features discordant *HNF1A* alleles in Norwegian Registry

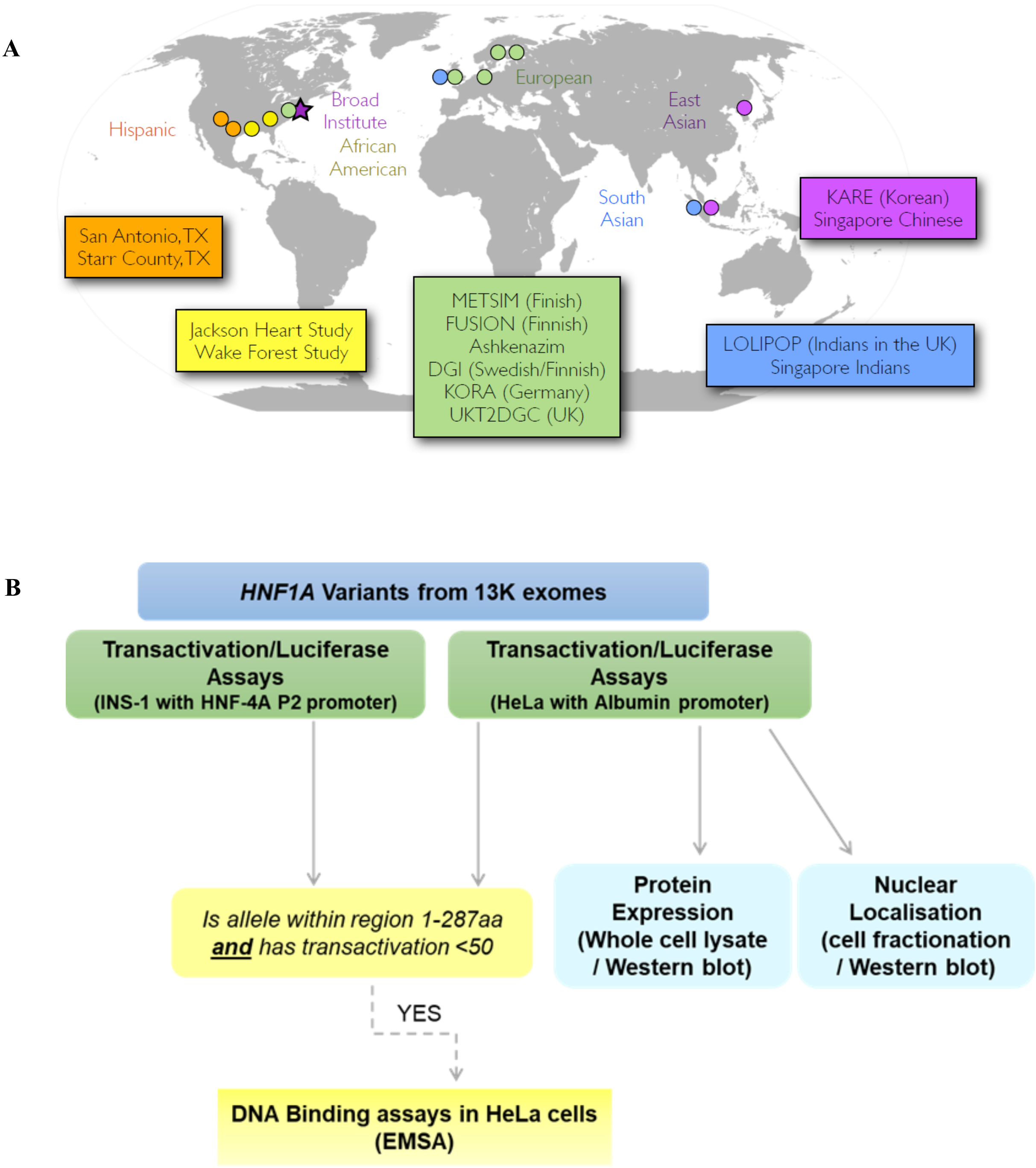

Figure S1. Ethnic Composition of a Multi-Consortia Led Exome Sequencing Study

(A). Molecular characterisation pipeline adopted by research groups at Oxford and Bergen to annotate the function of exome-detected *HNF1A* missense variants (B). Exome sequence data was generated from ~13K individuals (6,504 type 2 diabetes cases and 6,436 controls) from five ancestry groups with 82x mean coverage across the protein coding sequence of 18,281 genes, identifying 3.04 million variants (1.19 million protein-altering).[15]

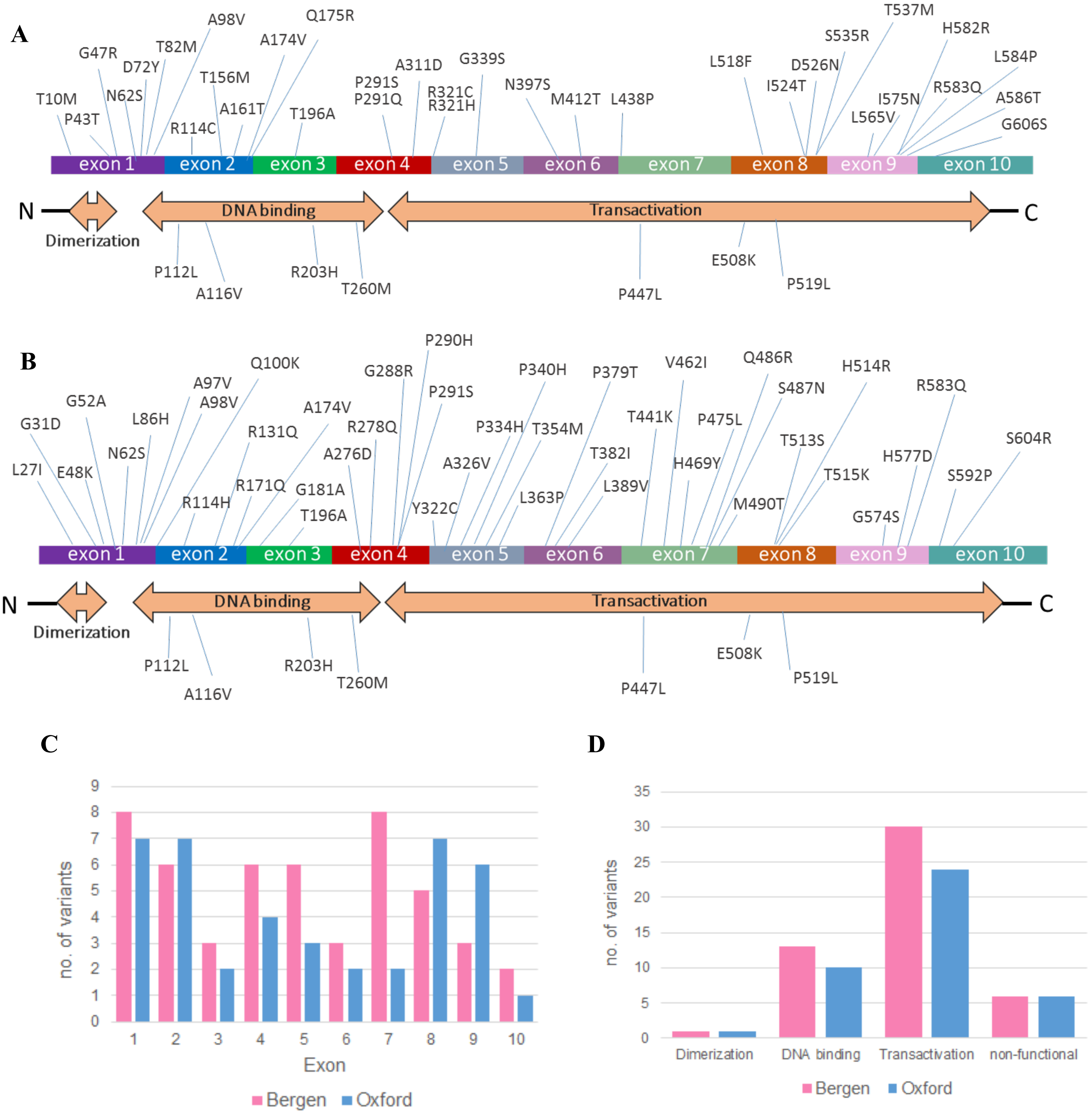

Figure S2. Division of Exome-Identified Non-Synonymous Missense Alleles in *HNF1A* Between Oxford and Bergen for Large-Scale Collaborative Functional Follow-up(A) Oxford variant set **(B)** Bergen variant set **(C)** Distribution of exome-detected *HNF1A* variants by centre based on exon position **(D)** Distribution of exome-detected *HNF1A* alleles by centre based on functional domain.

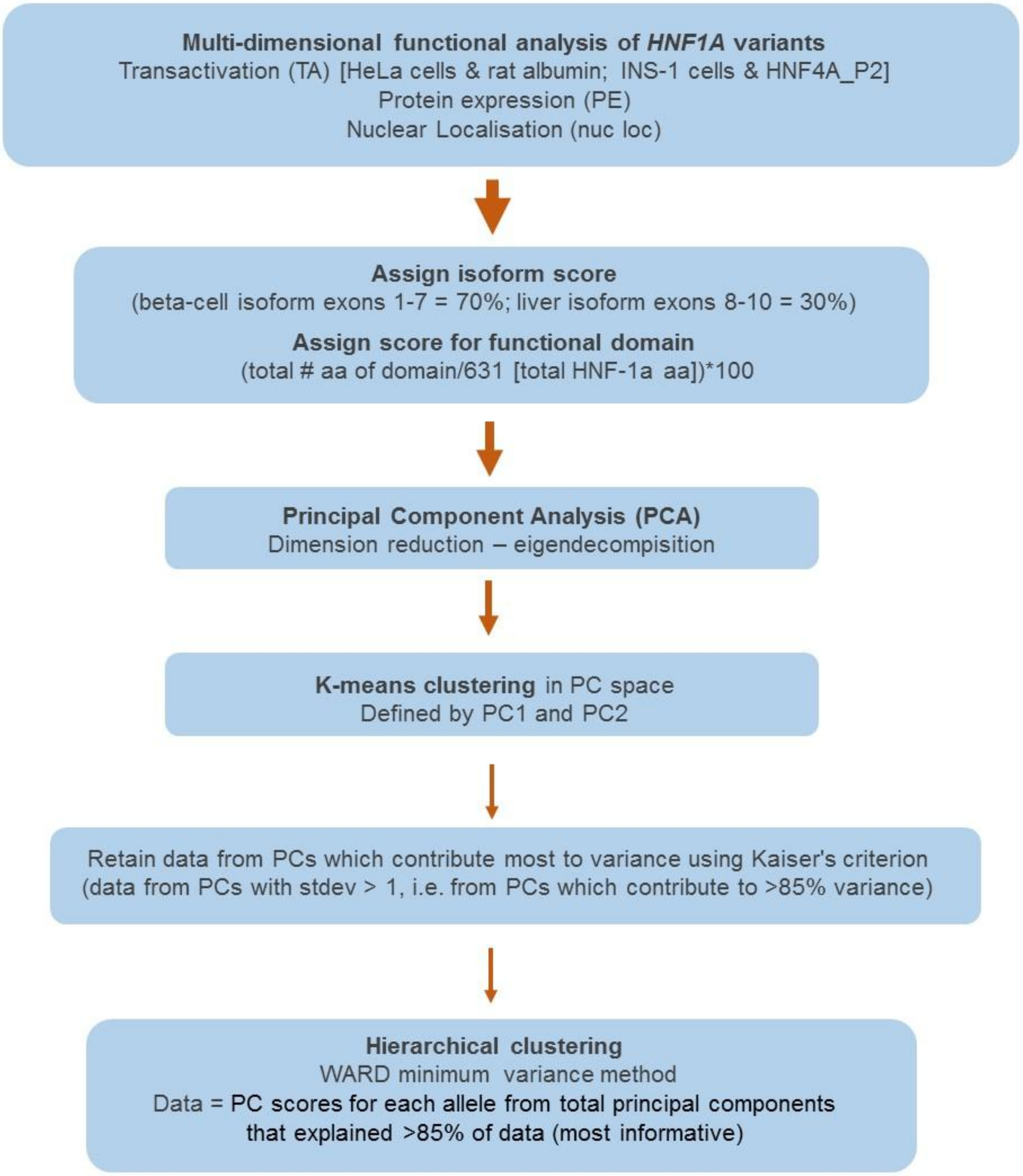

Figure S3. Analytical pipeline for unsupervised stratification of HNF1A missense variants along the in vivo glycaemic spectrum using multi-dimensional in vitro functional data. The pipeline we developed to perform unbiased classification of functionally characterised missense alleles in HNF1A is as follows: 1) dataset preparation (omitting missing values and unevenly represented functional parameters, and selecting the optimal input data format), 2) assignment of weights for tissue-specific isoform expression (liver v pancreatic beta-cell dominant isoforms) and functional domain (dimerization, undefined, DNA binding, transactivation), 3) Dimension reduction by principal component analysis, 4) retention of values from principal components which contribute the most to data variance according to Kaiser's criterion (>85% explained variance by eigenvalues i.e. all PCs with standard deviation > 1) to avoid fitting noise, 5) hierarchical clustering analysis of data points from PCs retained in step 4 using WARD minimum variance method. Abbreviations: aa= amino acids; stdev = standard deviation; PCs = principal components.

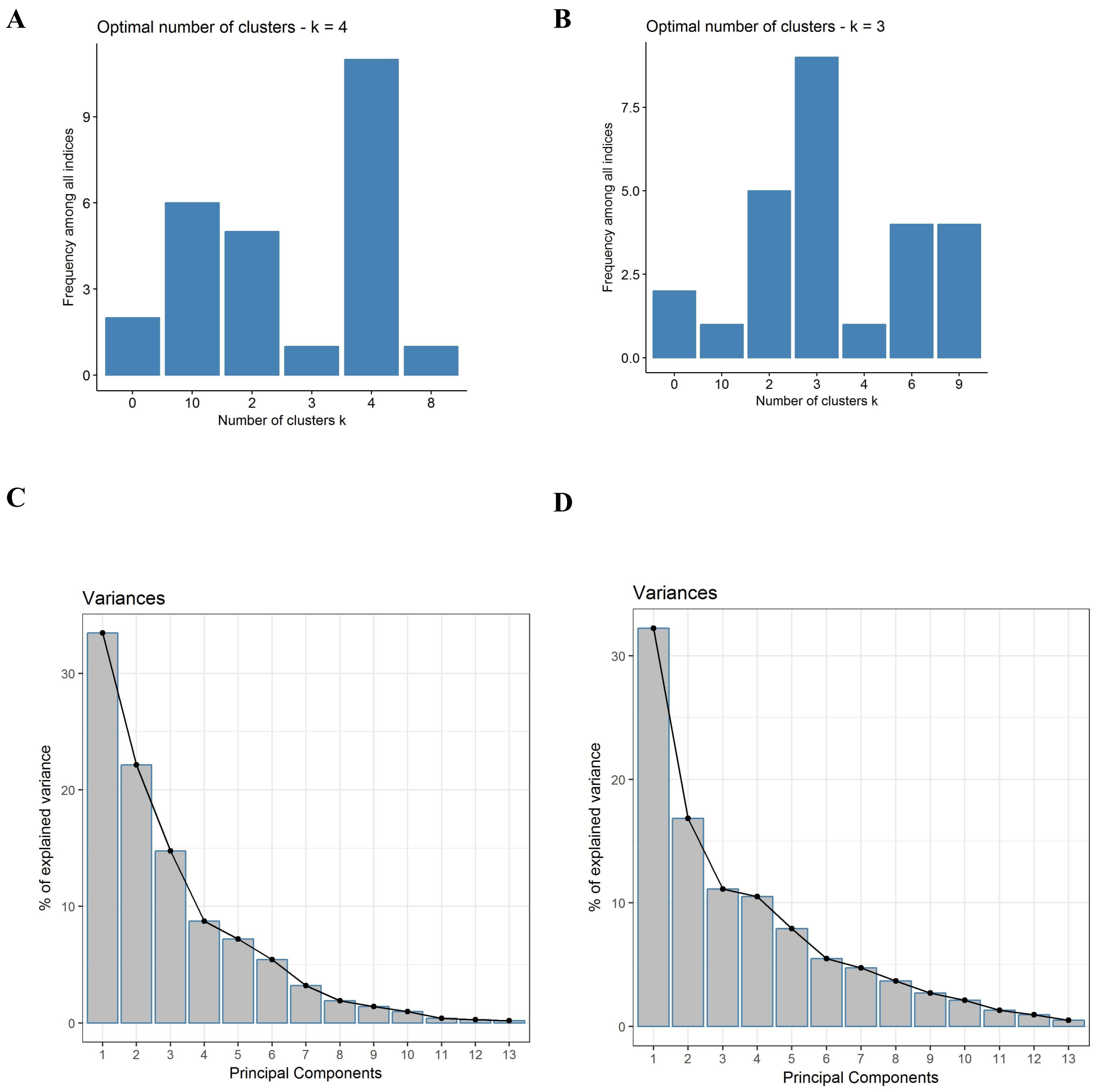

Figure S4. Assessment of the Optimal Number of Clusters to Use for Partitioning the Multi-Dimensional Datasets. Dataset generated at (A) Oxford and (B) Bergen. Best cluster scheme obtained using *NbClust* R package which provides majority rule across 30 tested indices using euclidean distance. **Scree plots showing percent explained variance from PCA of Oxford (C) and Bergen (D) datasets.**

Summary of Figure S5 and S6 - Functional studies of Protein Variants

The variants studied at both Centers included five exome variants (p.Asn62Ser, p.Ala174Val, p.Pro291Ser, p.Thr196Ala, p.Arg583Gln), two type 2 risk variants (p.Ala98Val, p.Glu508Lys), and six MODY variants (super controls; p.Ala116Val, p.Arg203His, p.Pro112Leu, p.Pro447Leu, p.Thr260Met, p.Pro519Leu), with single variants missing in some individual assays.

Correlation of functional effects between centers - For the transactivation assay, testing two promoters in different cell lines (HeLa/INS), the control variants correlated well between centers by range of effect; the MODY control variants presented the most severe effect, and the type 2 diabetes risk variants medium effects (deviation by the p.Ala116Val MODY control variant in one promoter assay). For the exome variants, the functional effect also correlated between centers, i.e. less severe effect than the rare type 2 risk variant (p.Glu508Lys) (deviation p.Pro291Ser between Centers). Overall, values of all tested variants were somewhat lower at one center (Bergen).

For the subsequent DNA binding assay, only one mutual exome variant was tested at both centers (p.Ala174Val), revealing correlating effects between the two centers. The same applied to the two MODY variants, although lower values (more severe effect) were detected at one center (Oxford).

For the protein expression assay, the MODY and type 2 risk variants correlated well between the centers (deviation for p.Glu508Lys exhibiting stronger reducing effect by one center (Bergen <0.6 fold versus Oxford WT-like). Two (p.Asn62Ser, p.Arg583Gln) of the five exome variants also deviated between the two centers (Oxford ~0.6 fold versus Bergen WT-like).

The nuclear localization assay correlated less between the centers. Two (p.Pro447Leu, p.Ala116Val) of six MODY variants, the two type 2 risk variants (p.Ala98Val, p.Glu508Lys), and the two exome variants (p.Ala174Val, p.Arg583Gln) did not correlate by range of effect (Bergen by stronger reducing effect compared to Oxford). The non-patient related control variant included as the best indicator of strongly reduced nuclear localization (DelB) did, however, correlate well between centers.

Of the four functional assays, it was not unexpected that functional effect between centers correlated best for the transactivation and DNA binding assays, whereas the protein expression and nuclear localization assays had more variable effect sizes when compared. This latter is most likely due several experimental steps prior to final quantifications, which is based on immunoblotting and antibody specificity.

A

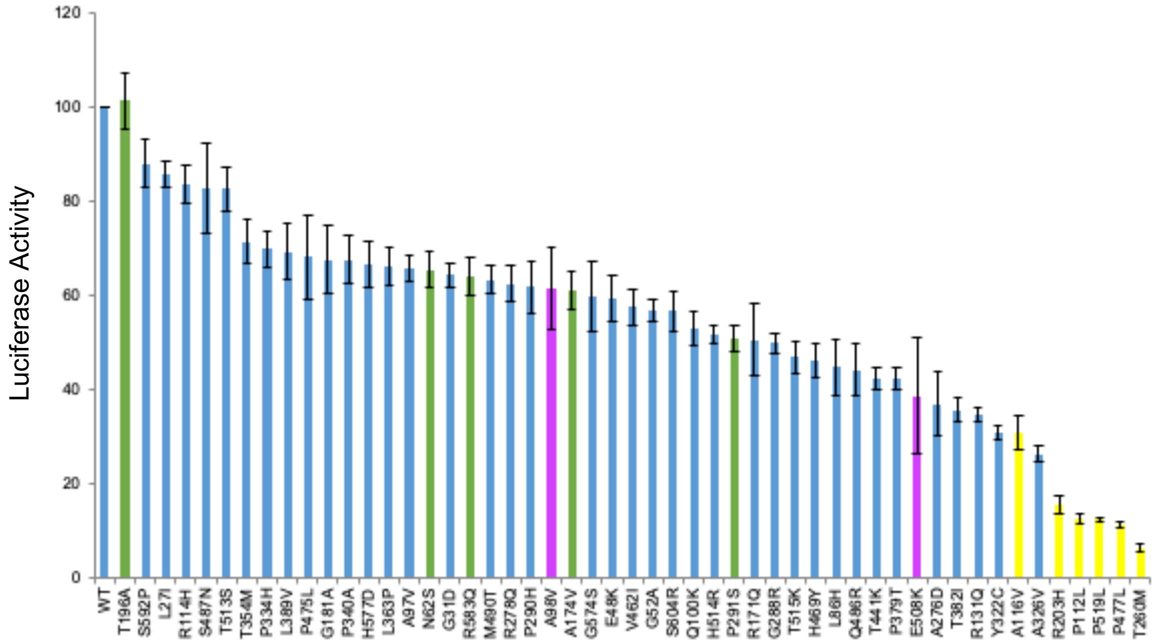
Luciferase Activity
120
100
80
60
40
20
0
WT T196A S592P L27I R114H S487N T513S T354M P334H L389V P475L G181A P340A H577D L363P A97V N62S G31D R583Q M490T R278Q P290H A98V A174V G574S E48K V462I G52A S604R Q100K H514R P291S R171Q G288R T515K H469Y L86H Q486R T441K P379T E508K A276D T382I R131Q Y322C A116V A326V R203H P112L P519L P477L T260M

B

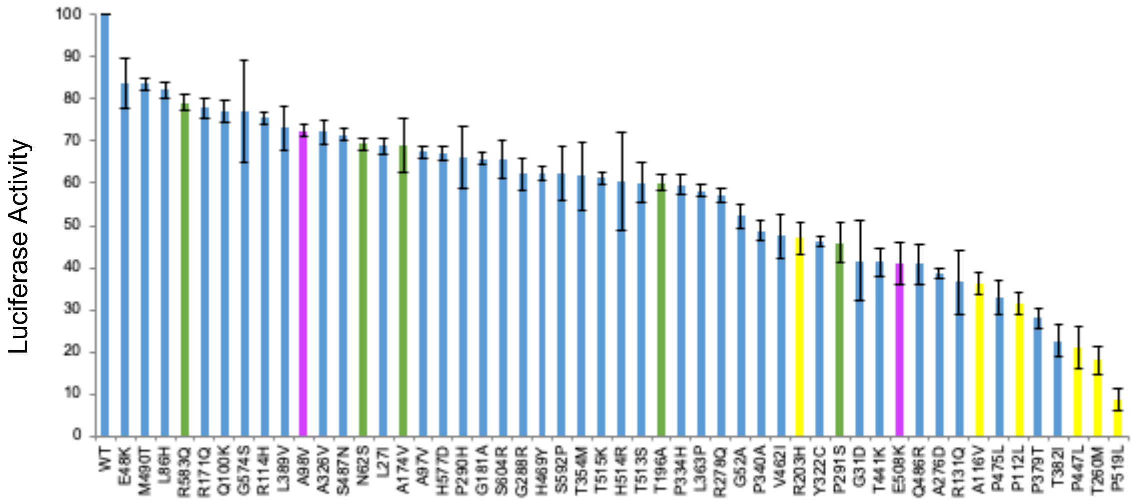
Luciferase Activity
100
90
80
70
60
50
40
30
20
10
0
WT E48K M490T L86H R583Q R171Q Q100K G574S R114H L389V A98V A326V S487N N62S L27I A174V A97V H577D P290H G181A S604R G288R H469Y S592P T354M T515K H514R T513S T196A P334H L363P R278Q G52A P340A V462I R203H Y322C P291S G31D T441K E508K Q486R A276D R131Q A116V P475L P112L P379T T382I P447L T260M P519L

C

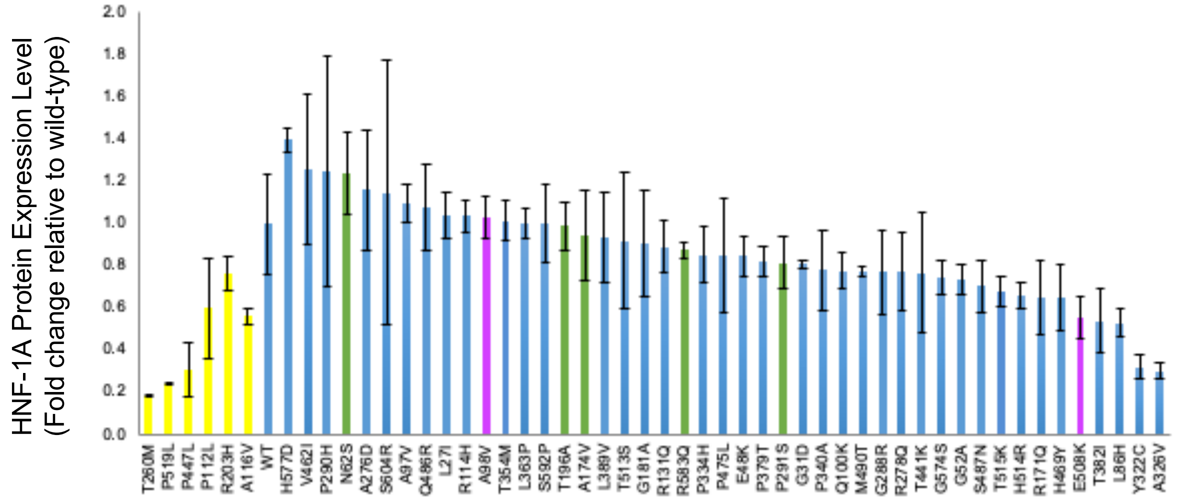
HNF-1A Protein Expression Level
(Fold change relative to wild-type)
2.0
1.8
1.6
1.4
1.2
1.0
0.8
0.6
0.4
0.2
0.0
T260M
P519L
P447L
P112L
R203H
A116V
WT
H577D
V462I
P290H
N62S
A276D
S604R
A97V
Q486R
L27I
R114H
A98V
T354M
L363P
S592P
T196A
A174V
L389V
T513S
G181A
R131Q
R583Q
P334H
P475L
E48K
P379T
P291S
G31D
P340A
Q100K
M490T
G288R
R278Q
T441K
G574S
G52A
S487N
T515K
H514R
R171Q
H469Y
E508K
T382I
L86H
Y322C
A326V

D

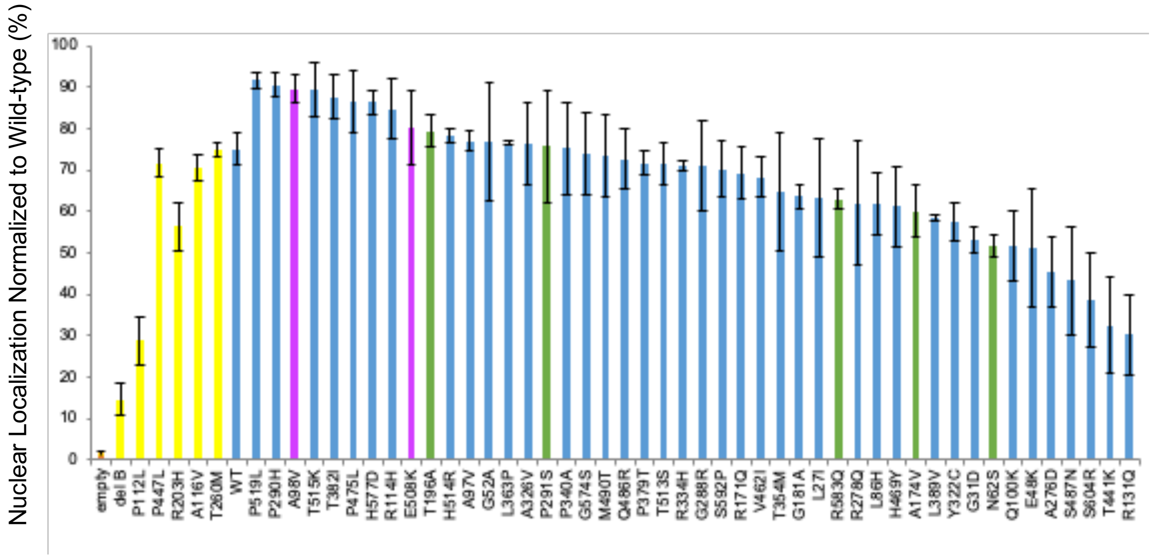
Nuclear Localization Normalized to Wild-type (%)
100
90
80
70
60
50
40
30
20
10
0
empty
del B
P112L
P447L
R203H
A116V
T260M
WT
P519L
P290H
A98V
T515K
T382I
P475L
H577D
R114H
E508K
T196A
H514R
A97V
G52A
L363P
A326V
P291S
P340A
G574S
M490T
Q486R
P379T
T513S
R334H
G288R
S592P
R171Q
V462I
T354M
G181A
L27I
R583Q
R278Q
L86H
H469Y
A174V
L389V
Y322C
G31D
N62S
Q100K
E48K
A276D
S487N
S604R
T441K
R131Q

E

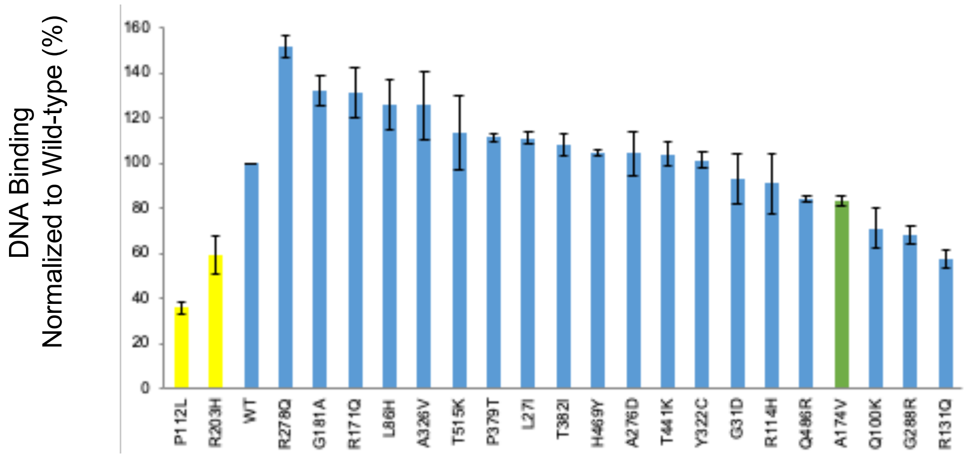

Figure S5. Functional Studies of Protein Variants (Bergen)

A and B) Transcriptional activity of HNF-1A protein variants measured by luciferase reporter assay. Transcriptional activity was performed in two individual cell lines using two different promoter-linked luciferase reporter constructs. A) HeLa cell line using rat albumin promoter and B) INS-1 cell line using HNF4AP2 promoter. Cells were transiently transfected with wild-type or variant *HNF1A* variant plasmids together with reporter plasmids pGL3-RA (Firefly Luciferase under rat albumin promoter) or pGL3-HNF4AP2 (Firefly Luciferase under HNF4AP2 promoter), and pRL-SV40 (Renilla Luciferase as internal control). Cells were harvested/lysed 24 h after transfection and Firefly activity recorded after normalizing for Renilla activity. HNF-1A variant measurements are given in percentage of wild-type activity (100%). Each bar represents the mean of nine readings ± SD; three parallel readings were conducted on each of 3 experimental days (n = 3). Different colors are used for different group of variants; shared control variants are shown in green, type 2 diabetes associated variants in pink and MODY control variants in yellow.

C) Variant effect on HNF-1A protein expression level. The level of expression of HNF-1A variant proteins was assessed relative to wild-type protein level in cells. For this purpose, 20 µl HeLa cell lysates prepared for transactivation assay (panel A) was analyzed by SDS-PAGE and immunoblotting using an HNF-1A specific antibody. A house-keeping protein (actin) was used as internal control for normalization and bands were quantitated using densitometric analysis by Quantity One 1-D software. Each bar represents the level of HNF-1A protein expression normalized to wild-type HNF-1A expression level (100%). Different colors are used for different group of variants; shared control variants are shown in green, type 2 diabetes associated variants in pink and MODY control variants in yellow.

D) Nuclear localization of HNF-1A variant proteins. The effect of individual *HNF1A* variants on HNF-1A protein localization (nuclear versus cytosolic) was assessed in cells. For this purpose, HeLa cells were cultured and transiently transfected with wild-type or *HNF1A* variant plasmids and sequential cell fractionation was performed 24h post-transfection by isolating the nuclear and cytosol fractions from each transfected sample. 20 µg total protein from each isolated compartment was analyzed for HNF-1A expression by SDS-PAGE and immunoblotting using an HNF-1A specific antibody. HNF-1A variant p.delB was included as a positive control for impaired nuclear localization (cytosolic retention). HNF-1A variant nuclear localization measurements are given in percentage of wild-type localization. Each bar represents the mean of nuclear localization of HNF-1A protein of two biological replicates in two experimental days (n = 2). Different colors are used for different group of variants; shared control variants are shown in green, type 2 diabetes associated variants in pink and MODY control variants in yellow.

E) DNA binding of HNF-1A protein variants to the rat albumin promoter as studied by EMSA. DNA binding ability test was conducted for HNF-1A variants that are either located in DNA binding domain (1-287 aa) or those that demonstrated transactivation activity < 50%. Electrophoretic mobility shift assay (EMSA) was performed to investigate the DNA binding ability of equal amounts of *in vitro*-synthesized HNF-1A proteins to a ^{32}P-radiolabeled rat albumin oligonucleotide containing an HNF-1A binding site. A coupled *in vitro* transcription and translation system (TNT) was used for expression of HNF-1A proteins. DNA-protein bound complexes were analyzed by DNA retardation gel (6%) electrophoresis followed by autoradiography. Level of DNA binding was obtained by quantification of the intensity of HNF-1A protein-oligonucleotide complexes. Measurements are given in percentage of wild-type binding activity (100%). Two *HNF1A*-MODY3 variants were used as control for low binding (yellow) and shared control variants are presented in green color.

A

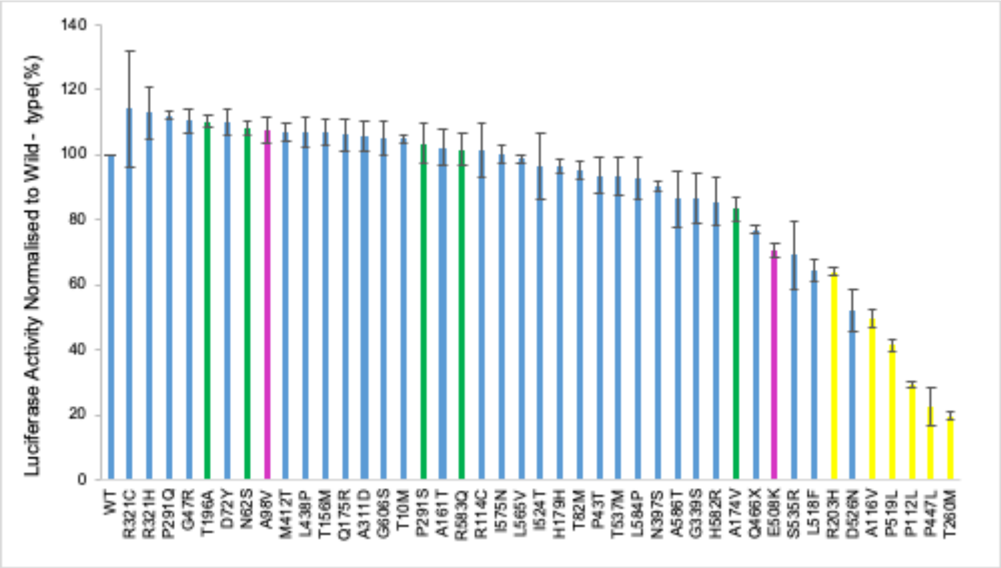
Luciferase Activity Normalised to Wild - type(%)
140
120
100
80
60
40
20
0
WT
R321C
R321H
P291Q
G47R
T196A
D72Y
N62S
A98V
M412T
L438P
T156M
Q175R
A311D
G606S
T10M
P291S
A161T
R583Q
R114C
I575N
L565V
I524T
H179H
T82M
P43T
T537M
L584P
N397S
A586T
G339S
H582R
A174V
Q466X
E508K
S535R
L518F
R203H
D526N
A116V
P519L
P112L
P447L
T260M

B

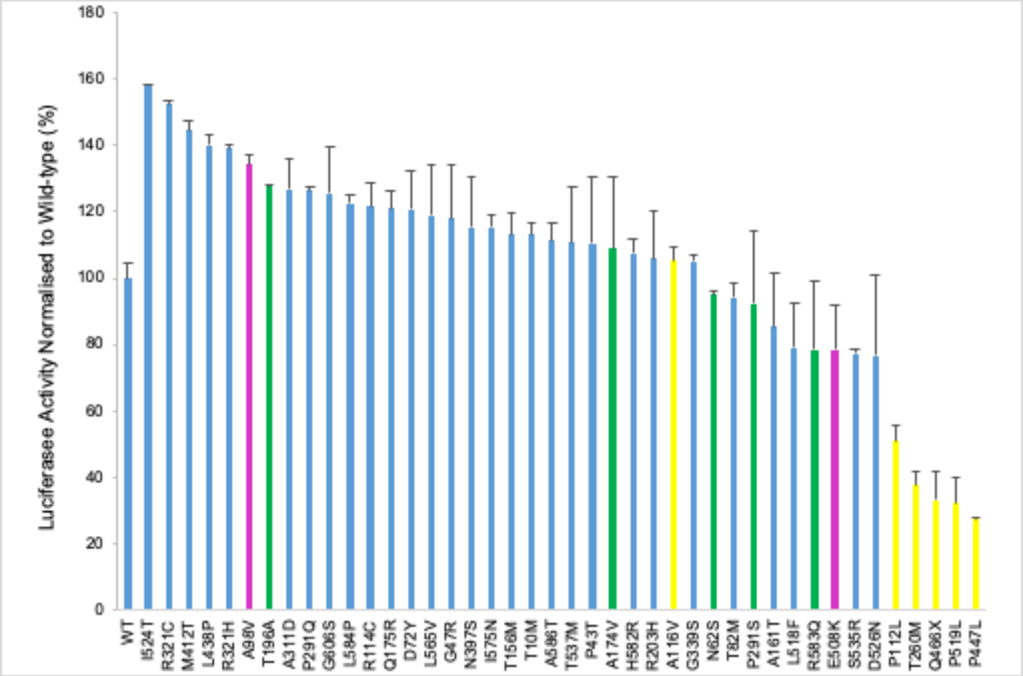
Luciferasee Activity Normalised to Wild-type (%)
180
160
140
120
100
80
60
40
20
0
WT
I524T
R321C
M412T
L438P
R321H
A98V
T196A
A311D
P291Q
G606S
L584P
R114C
Q175R
D72Y
L565V
G47R
N397S
I575N
T156M
T10M
A586T
T537M
P43T
A174V
H582R
R203H
A116V
G339S
N62S
T82M
P291S
A161T
L518F
R583Q
E508K
S535R
D526N
P112L
T260M
Q466X
P519L
P447L

C

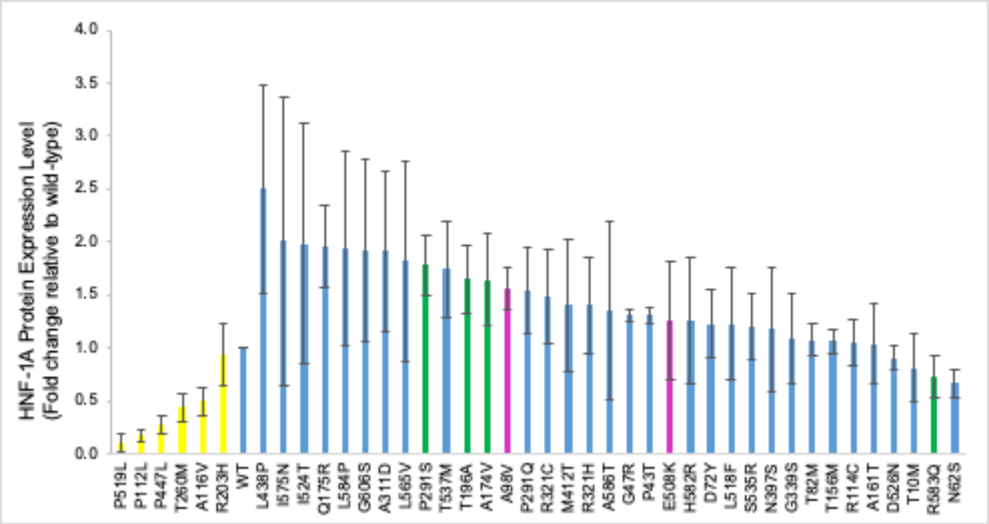
HNF-1A Protein Expression Level
(Fold change relative to wild-type)
4.0
3.5
3.0
2.5
2.0
1.5
1.0
0.5
0.0
P519L
P112L
P447L
T260M
A116V
R203H
WT
L438P
I575N
I524T
Q175R
L584P
G606S
A311D
L565V
P291S
T537M
T196A
A174V
A98V
P291Q
R321C
M412T
R321H
A586T
G47R
P43T
E508K
H582R
D72Y
L518F
S535R
N397S
G339S
T82M
T156M
R114C
A161T
D526N
T10M
R583Q
N62S

D

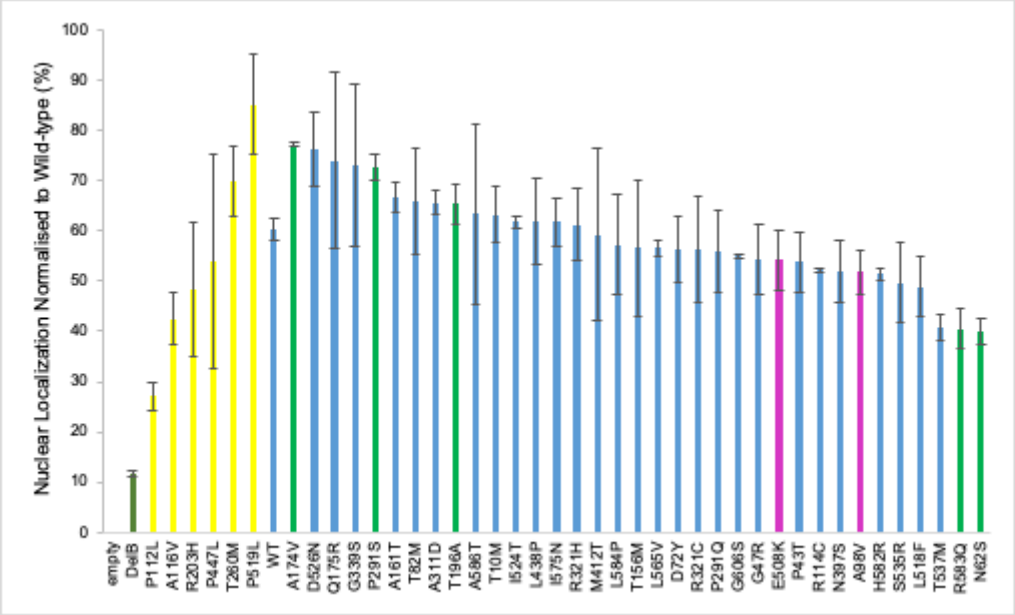
Nuclear Localization Normalised to Wild-type (%)
100
90
80
70
60
50
40
30
20
10
0
empty
DelB
P112L
A116V
R203H
P447L
T260M
P519L
WT
A174V
D526N
Q175R
G339S
P291S
A161T
T82M
A311D
T196A
A586T
T10M
I524T
L438P
I575N
R321H
M412T
L584P
T156M
L565V
D72Y
R321C
P291Q
G606S
G47R
E508K
P43T
R114C
N397S
A98V
H582R
S535R
L518F
T537M
R583Q
N62S

E

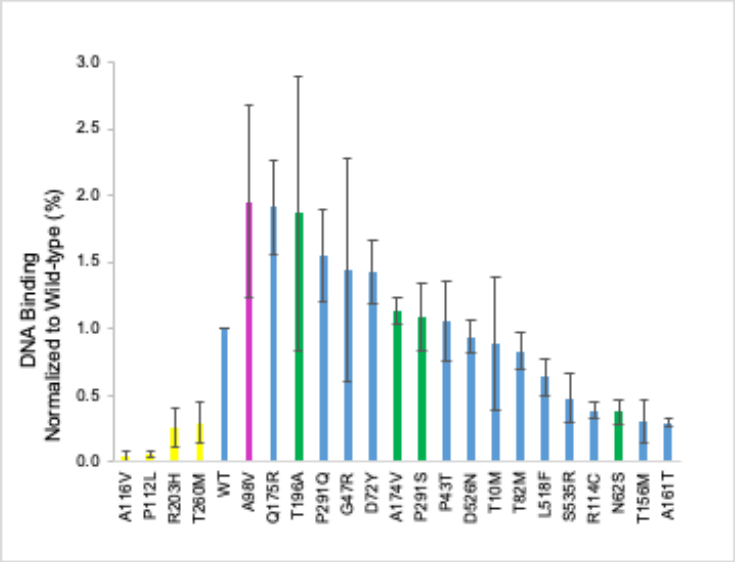

Figure S6. Functional Studies of Protein Variants (Oxford)

A and B) Transcriptional activity of HNF-1A protein variants measured by luciferase reporter assay. Transcriptional activity was performed in two individual cell lines using two different promoter-linked luciferase reporter constructs. A) HeLa cell line using rat albumin promoter and B) INS-1 cell line using *HNF4A* P2 promoter. Cells were transiently transfected with wild-type or variant *HNF1A* variant plasmids together with reporter plasmids pGL3-RA (Firefly Luciferase under rat albumin promoter) or pGL3-*HNF4A* P2 (Firefly Luciferase under *HNF4A* P2 promoter), and pRL-SV40 (Renilla Luciferase as internal control). Cells were harvested/lysed 24 h after transfection and Firefly activity recorded after normalizing for Renilla activity. HNF-1A variant measurements are given in percentage of wild-type activity (100%). Each bar represents the mean of nine readings ± SD; three parallel readings were conducted on each of three experimental days (n = 3). Different colors are used for different group of variants; shared control variants are shown in green, type 2 diabetes associated variants in pink and MODY control variants in yellow.

C) Variant effect on HNF-1A protein expression level. The level of expression of HNF-1A variant proteins was assessed relative to wild-type protein level in cells. For this purpose, HeLa cells were cultured and transiently transfected with wild-type or *HNF1A* variant plasmids. Cells were harvested at 24 hrs and aliquoted prior to lysing for the protein expression levels and EMSA or nuclear localization. 10 µg of HeLa cell lysates prepared for transactivation assay (Figure A) was analyzed by SDS-PAGE and immunobloting using an HNF-1A specific antibody. A house-keeping protein (B-tubulin) was used as internal control for normalization of levels of tubulin versus HNF-1A specific bands., using densitometric analysis by Image Lab software (Bio-Rad). Each bar represents the level of HNF-1A protein expression normalized to wild-type HNF-1A expression level (100%). Different colors are used for different groups of variants; shared control variants are shown in green, type 2 diabetes associated variants in pink and MODY control variants in yellow.

D) Nuclear localization of HNF-1A variant proteins. The effect of individual *HNF1A* variants on HNF-1A protein localization (nuclear versus cytosolic) was assessed in cells. For this purpose, an aliquot of the HeLa cells that had been transfected and harvested at 24hr were lysed to isolate the nuclear and cytosol fraction. 20 µg total protein from each isolated compartment was analyzed for HNF-1A expression by SDS-PAGE and immunobloting using an HNF-1A specific antibody. HNF-1A variant p.delB was included as a positive control for impaired nuclear localization (cytosolic retention). HNF-1A variant nuclear localization measurements are given in percentage of wild-type localization. Each bar represents the mean of nuclear

localization of HNF-1A protein of two biological replicates in two experimental days (*n* = 2). Different colors are used for different groups of variants; shared control variants are shown in green, type 2 diabetes associated variants in pink and MODY control variants in yellow.

E) DNA binding of HNF-1A protein variants to the rat albumin promoter as studied by EMSA. DNA binding ability test was conducted for *HNF1A* variants that are either located in DNA binding domain (1-287 aa) or those that demonstrated transactivation activity < 50%. Electrophoretic mobility shift assay (EMSA) was performed using an aliquot of the lysed HeLa cells used for the protein expression levels. The HNF-1a wild-type or variant protein was bound to a fluorescently labelled probe and then separated using a TBE polyacrylamide gel. An HNF-1a antibody was used to generate a super-shift with an aliquot of the wild-type protein, prior to the probe binding. Measurements are given as percentage of wild-type binding activity (100%). Different colors are used for different groups of variants; shared control variants are shown in green, type 2 diabetes associated variants in pink and MODY control variants in yellow.

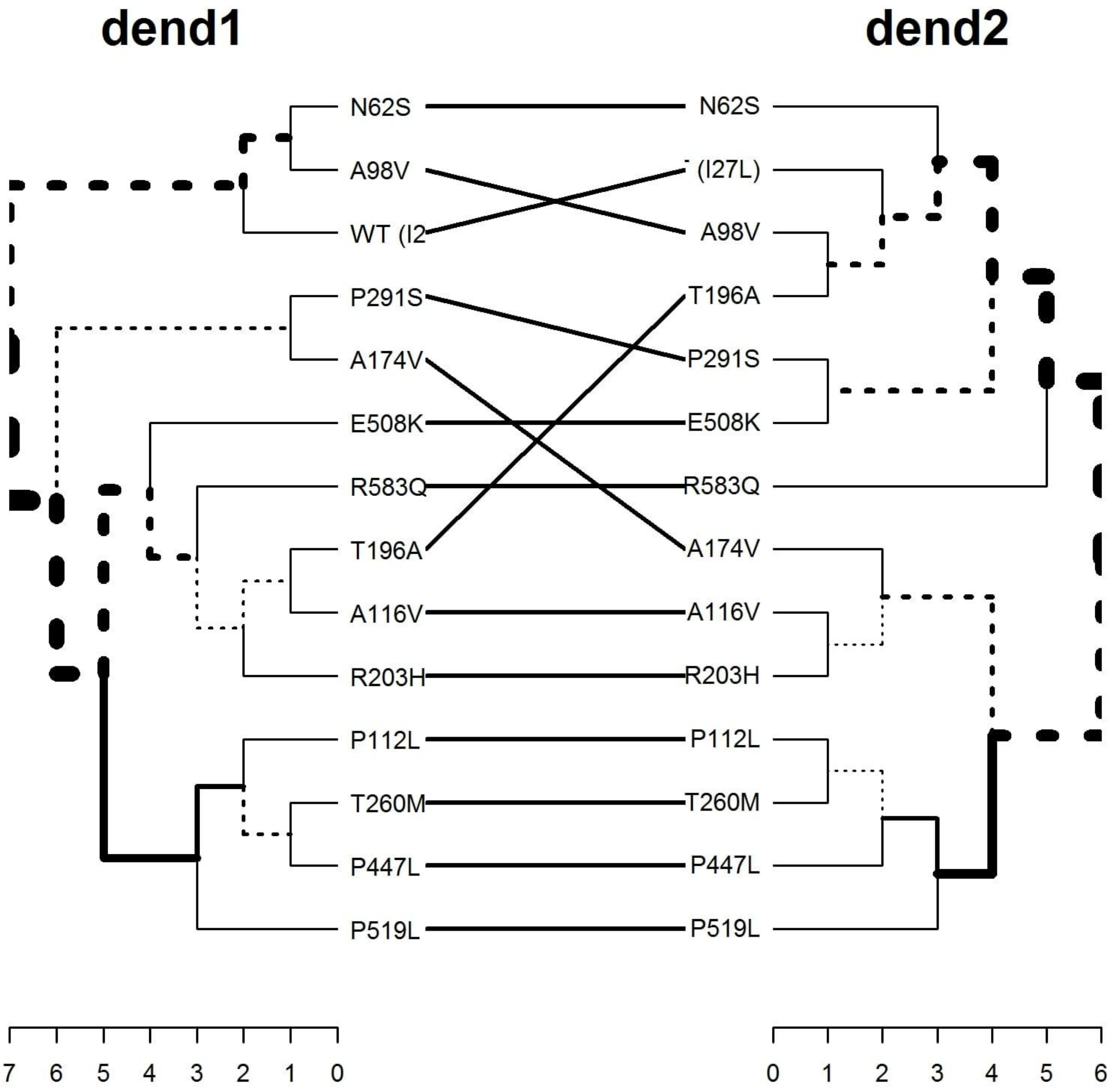

Figure S7. Clustering Alignment of *HNF1A* Missense Variants Shared Between Both Centres at Oxford (dend1and Bergen (dend2). Entanglement (quality of alignment score score from 1 to 0 where lower values = good alignment quality) = 0.055. Dashed lines highlight nodes which contain a combination of alleles not present in the other tree (thickness corresponds to height). The connecting lines highlight subgroups and/or cluster members that are present in both dendrograms. Rotational properties maintained from full variant set dendrogams shown in Figure 3.

Table S1. Exome-Detected *HNF1A* Missense Alleles Selected for Functional Follow-Up (*n* = 73)

(Excel file)

HNF1A reference sequence = NM_000545.6. Colour coding of first three columns (Variant and CDS position NM_0005454): blue = Oxford variant set; red = Bergen variant set; gray = variants shared and characterised by both research groups at Oxford and Bergen. Green highlights variants identified with a prevalence greater than 0.

Table S2. MODY Reference Alleles (*n* = 6)

(Excel file)

Table S3. Scores for Tissue Specific Isoform Expression and Functional Domain

Isoform	Functional Domain
Pancreatic beta-cell isoforms<br>*HNF1A(A)*: exons 1-6<br>UniProtKB Protein isoform identifier P208231-3<br><br>*HNF1A(B)*: exons 1-7<br>UniProtKB Protein isoform identifier P208231-2<br><br>***Hepatic isoform (full length gene)***<br>*HNF1A(C):* exons 1-10<br>NCBI Reference Sequence: NM_000545.8<br>UniProtKB Protein isoform identifier P208231-1	***HNF-1A functional domains***<br>Dimerization = 32 amino acids<br>Undefined = 66 amino acids<br>DNA binding = 187 amino acids<br>Transactivation = 343 amino acids
Weight (%)<br><br>Variants in *HNF1A(A)* and *HNF1A(B)* = [(no. of exons in predominant beta-cell isoforms) ÷ 10 (no. of exons in full length *HNF1A*)] × 100 = 70%<br><br>Variants exclusively present in *HNF1A(C)* = [(no. of additional exons present exclusively in predominant liver isoform) ÷ 10 (no. of exons in full length *HNF1A*)] × 100 = 30%	***Weight (%)***<br><br>[no. of amino acids of domain in which allele is expressed ÷ amino acid length of HNF-1a] × 100<br><br>Dimerization = 4.9%<br>Undefined = 10.45%<br>DNA binding = 29.6%<br>Transactivation = 54.3%

Table S4. Features of *HNF1A* Allele Carriers Documented in UK Registry

Features	Measure
Age at diagnosis	age (years)
Initial Treatment	Diet/OHA/Insulin
Current Treatment	Diet/OHA/Insulin
Sulphonylurea sensitivity	Yes/No
BMI	BMI (kg/m^2)
HbA1c	%
GAD	U/mL
ZnT8	U/mL
IA2	U/mL
C-Peptide	pmol/L
No of generations DM	number of generations with diabetes
Mother DM	Yes/No
Father DM	Yes/No
No of Children DM	number of children with diabetes
Ethnic Origin	ethnicity
Referred to clinic for MODY testing	Yes/No

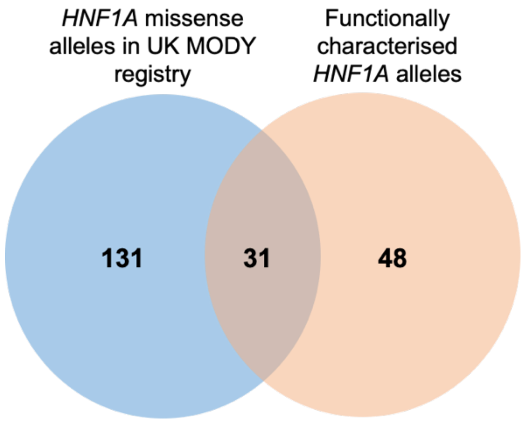

GAD, ICA, IA2, ZnT8 = islet autoantibodies; UCPCR = urinary C-peptide creatinine ratio; OHA = oral hypoglycaemic agent; BMI = body mass index; DM= diabetes mellitus; HbA1c = glycated hemoglobin.

Table S5. Features of *HNF1A* Allele Carriers Documented in Norwegian Registry

Features	Measure
Age at diagnosis	age (years)
Initial Treatment	Diet/OHA/Insulin
Current Treatment	Diet/OHA/Insulin
Sulphonylurea sensitivity	Yes/No
BMI	BMI (kg/m^2)
HbA1c	%
GAD -ve	Yes/No
GAD +ve	Yes/No
ICA +ve	Yes/No
ICA -ve	Yes/No
ZnT8 -ve	Yes/No
ZnT8 +ve	Yes/No
IA2 -ve	Yes/No
IA2 +ve	Yes/No
UCPCR Value	nmol/mmol
C-Peptide	pmol/L
No of generations DM	number of generations with diabetes
Mother DM	Yes/No
Father DM	Yes/No
No of Children DM	number of children with diabetes
Ethnic Origin	ethnicity

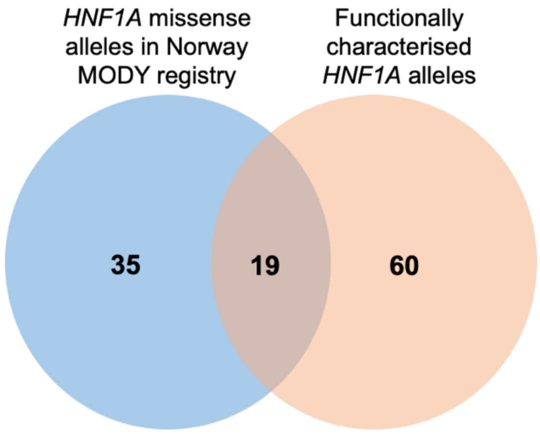

GAD, IA2, ZnT8 = islet autoantibodies; OHA = oral hypoglycemic agent; BMI = body mass index; DM= diabetes mellitus; HbA1c = glycated hemoglobin. Common alleles (AF 3-35%) indicated by a (*).

Table S6. Clinical Features Associated with Functionally-Clinically Discordant *HNF1A* Alleles in UK Registry

(Excel file)

Clinical features and additional information available on each of the listed alleles from the UK MODY diagnostic registry. With the exception of p.R131Q (*n* = 4 documented cases; information displayed as range across the four), details are associated with a single case. Abbreviations: NGS = next generation sequencing; dx = diagnosis; SU = sulfonylurea; FH = family history; BMI = body mass index; HbA1c = glycated hemoglobin.

Table S7. Clinical Features Associated with Functionally-Clinically Discordant *HNF1A* Alleles in Norwegian Registry

(Excel file)

Clinical features and additional information available on each of the listed alleles from the Norway MODY diagnostic registry. Abbreviations: NGS = next generation sequencing; dx = diagnosis; SU = sulfonylurea; FH = family history; BMI = body mass index; HbA1c = glycated hemoglobin
