# Supplementary material for: Unsupervised Clustering of Missense Variants in HNF1A Using Multidimensional Functional Data Aids Clinical Interpretation
Source: Am J Hum Genet. 2020 Sep 9;107(4):670–82. doi: 10.1016/j.ajhg.2020.08.016 (PMC7536579; doi:10.1016/j.ajhg.2020.08.016)
Supplement: Document S2. Article plus Supplemental Information [file mmc6.pdf]

ARTICLE

Unsupervised Clustering of Missense Variants in *HNF1A* Using Multidimensional Functional Data Aids Clinical Interpretation

Sara Althari,[1,17] Laeya A. Najmi,[2,3,4,17] Amanda J. Bennett,[1] Ingvild Aukrust,[2,3] Jana K. Rundle,[1] Kevin Colclough,[5,6] Janne Molnes,[2,3] Alba Kaci,[2,7] Sameena Nawaz,[1] Timme van der Lugt,[8,15] Neelam Hassanali,[1] Anubha Mahajan,[1,10] Anders Molven,[2,9,11] Sian Ellard,[5,6] Mark I. McCarthy,[1,10,12,16] Lise Bjørkhaug,[13] Pål Rasmus Njølstad,[2,7,18,*] and Anna L. Gloyn[1,10,12,14,18]

Summary

Exome sequencing in diabetes presents a diagnostic challenge because depending on frequency, functional impact, and genomic and environmental contexts, *HNF1A* variants can cause maturity-onset diabetes of the young (MODY), increase type 2 diabetes risk, or be benign. A correct diagnosis matters as it informs on treatment, progression, and family risk. We describe a multi-dimensional functional dataset of 73 *HNF1A* missense variants identified in exomes of 12,940 individuals. Our aim was to develop an analytical framework for stratifying variants along the *HNF1A* phenotypic continuum to facilitate diagnostic interpretation. *HNF1A* variant function was determined by four different molecular assays. Structure of the multi-dimensional dataset was explored using principal component analysis, k-means, and hierarchical clustering. Weights for tissue-specific isoform expression and functional domain were integrated. Functionally annotated variant subgroups were used to re-evaluate genetic diagnoses in national MODY diagnostic registries. *HNF1A* variants demonstrated a range of behaviors across the assays. The structure of the multi-parametric data was shaped primarily by transactivation. Using unsupervised learning methods, we obtained high-resolution functional clusters of the variants that separated known causal MODY variants from benign and type 2 diabetes risk variants and led to reclassification of 4% and 9% of *HNF1A* variants identified in the UK and Norway MODY diagnostic registries, respectively. Our proof-of-principle analyses facilitated informative stratification of *HNF1A* variants along the continuum, allowing improved evaluation of clinical significance, management, and precision medicine in diabetes clinics. Transcriptional activity appears a superior readout supporting pursuit of transactivation-centric experimental designs for high-throughput functional screens.

Introduction

Precision medicine increasingly relies on an accurate interpretation of the consequence of genetic variation. Large-scale multi-ethnic genetic sequencing studies have challenged our understanding of the relationship between coding variants in Mendelian disease genes, including those involved in monogenic forms of diabetes such as *HNF1A* (MIM: 142410). Until relatively recently, the consensus has been that heterozygous highly penetrant loss-of-function alleles in *HNF1A* give rise to a clinically distinct diabetes subtype, characterized by an early age of onset (typically < 25 years), dominant inheritance, sensitivity to sulphonylureas, and non-obesity, and termed HNF1A maturity-onset diabetes of the young (HNF1A-MODY [MIM: 600496]).[1] While this genotype-phenotype correlation is true for a subset of *HNF1A* variant carriers, it represents one end of a broad spectrum of *HNF1A* variant effects.[2–4]

Genome-wide association and next-generation sequencing studies of randomly ascertained individuals have challenged binary assumptions and overinflated pathogenicity estimates regarding variants in *HNF1A* (and other Mendelian disease genes) and identified common coding variants of low effect associated with increased risk of type 2 diabetes (MIM: 125853).[5–8] Whole-exome sequencing studies in populations of Mexican American ancestry have revealed a low-frequency missense variant (c.1522G>A [p.Glu508Lys]) in *HNF1A* associated with a 5-fold increase in type 2 diabetes prevalence.[3] These complex genomic insights warrant a more nuanced understanding

[1]Oxford Centre for Diabetes, Endocrinology & Metabolism, University of Oxford, Oxford OX3 7LE, UK; [2]Center for Diabetes Research, Department of Clinical Science, University of Bergen, 5020 Bergen, Norway; [3]Department of Medical Genetics, Haukeland University Hospital, 5021 Bergen, Norway; [4]Department of Medicine, Division of Cardiovascular Medicine, Stanford University School of Medicine, Stanford, CA 94305-5101, USA; [5]Institute of Biomedical and Clinical Science, University of Exeter Medical School, Exeter EX1 2LU, UK; [6]Exeter Genomics Laboratory, Royal Devon and Exeter NHS Foundation Trust, Exeter EX2 5DW, UK; [7]Department of Pediatrics and Adolescents, Haukeland University Hospital, 5021 Bergen, Norway; [8]Hormone Laboratory, Haukeland University Hospital, 5021 Bergen, Norway; [9]Department of Clinical Medicine, University of Bergen, 5020 Bergen, Norway; [10]Wellcome Centre for Human Genetics, University of Oxford, Oxford OX3 7BN, UK; [11]Department of Pathology, Haukeland University Hospital, 5021 Bergen, Norway; [12]Oxford NIHR Biomedical Research Centre, Churchill Hospital, Oxford OX3 7LE, UK; [13]Department of Safety, Chemistry, and Biomedical Laboratory Sciences, Western Norway University of Applied Sciences, 5020 Bergen, Norway; [14]Division of Endocrinology, Department of Pediatrics, Stanford School of Medicine, Stanford University, Stanford, CA 94305-5101, USA
[15]Present address: Department of Pharmacology and Toxicology, Maastricht University, Maastricht 6211, the Netherlands
[16]Present address: Human Genetics, Genentech, South San Francisco, CA 94080, USA
[17]These authors contributed equally
[18]These authors contributed equally
*Correspondence: pal.njolstad@uib.no
https://doi.org/10.1016/j.ajhg.2020.08.016.
© 2020 The Authors. This is an open access article under the CC BY license (http://creativecommons.org/licenses/by/4.0/).

of the phenotypic manifestation of *HNF1A* gene variants: some alleles are sufficient for early-onset sulfonylurea-responsive diabetes (HNF1A-MODY), although it should be noted that not all carriers of these alleles get early-onset diabetes; not even diabetes at all. Moreover, some alleles modify susceptibility for developing complex multifactorial hyperglycemia later in life (type 2 diabetes), and most alleles will likely manifest as benign and neutral.

A correct diabetes diagnosis is important because a mutation in *HNF1A* leads to clinical actions involving diagnosis, treatment, and genetic counselling. Individuals with rare, deleterious *HNF1A* alleles and young-onset diabetes (typically < 25 years) are sensitive to treatment with oral sulfonylureas and can often avoid insulin injections until late in life.[9,10]

The ubiquity of genetic sequencing means that more novel and incidentally detected variants of uncertain clinical significance (VUS) will be identified in individuals with less extreme phenotypes.[11] The challenge today is in the ability to map *HNF1A* sequence-function relationships at high fidelity, using clinical and molecular characterization and analytical pipelines with sensitivity to capture the subtleties along the pathophysiological continuum. Rigorous functional follow-up of rare sequence-identified alleles in *HNF1A* is crucial to making correct assignments of pathogenicity. Indeed, functional data are considered a strong line of evidence for accurate clinical diagnostic classification of variants.[12] Furthermore, it has been shown that diabetes severity in *HNF1A* variant carriers is influenced by allele position in the gene: the transactivation domain is more tolerant to genetic variation and variants in the latter exons[8–10] are only present in hepatocyte-dominant isoforms and would thus not likely translate to a strong beta-cell phenotype.[13,14]

To understand the relationship between *HNF1A* sequence variation, molecular dysfunction, and clinical phenotype, we characterized the functional impact of a total of 73 *HNF1A* missense variants detected in the exomes of 12,940 multi-ethnic type 2 diabetes case subjects and control subjects using standard functional assays. Our primary objective was to develop an analytical approach that would enable (1) an unbiased and comprehensive evaluation of *HNF1A* variant behavior based on multiple molecular mechanisms and (2) sensitive mapping of multi-dimensional *in vitro* function to *HNF1A* glycemic phenotypes *in vivo*. We hypothesized that severity of molecular dysfunction *in vitro* (wild-type/wild-type-like, moderate/intermediate impact, loss-of-function/deleterious) would correlate positively with the severity of clinical phenotype (benign, increased type 2 diabetes risk, young-onset sulfonylurea-responsive hyperglycemia).

Material and Methods

The study was approved by the regional ethical committee in Bergen (#2009/2079). We investigated the function of all rare (MAF < 0.5%) and low-frequency (0.5% < MAF < . 5%) as well as three common (MAF > 5%) *HNF1A* nonsynonymous missense variants (n = 73) identified in an exome sequencing study of 12,940 type 2 diabetes case subjects and control subjects from five different ancestry groups[15] (Figure S1, Table S1). Collectively, the variants did not enrich for a type 2 diabetes phenotype under any of the several variant filters used (MAF < 0.1%, conserved and predicted damaging [PolyPhen: SKAT p value = 0.30, BURDEN p value = 0.37]).[15]

Bioinformatic Prediction

The following four *in silico* tools were used to evaluate the pathogenicity of the alleles: SIFT,[16] PolyPhen-2,[17] MutationTaster,[18] and Combined Annotation Dependent Depletion (CADD).[19] A CADD cut-off score of 15 was used (>15, pathogenic).

Functional Characterization

The individual effects of the 73 *HNF1A* missense variants were functionally investigated by two research teams at the Universities of Oxford (UK) and Bergen (Norway) using four different molecular assays (see detailed description of assays below). Using two laboratories allowed us to evaluate the robustness of the functional studies. Each laboratory assessed a unique set of exome-detected variants (n > 30), a shared subset of exome-detected variants (n = 5), shared type 2 diabetes risk variants (n = 2), as well as shared HNF1A-MODY reference variants (positive controls, n = 6) (Figures S2 and S3). The positive controls were selected on the basis of previously reported functional data supporting pathogenicity, clinical evidence for causality (sulfonyurea sensitivity in multiple carriers), and/or genetic (co-segregation) evidence to support their role pathogenesis (Table S2). Plasmid and HNF-1A variant constructs, transactivation assays, HNF-1A protein abundance, subcellular localization, and DNA binding are detailed below.

Plasmid and HNF-1A Variant Constructs

A construct encoding the human *HNF1A* cDNA (GenBank: NM_000545.6) in cDNA3.1 His/C plasmid was used as wild-type and template for introducing *HNF1A* variants using the QuikChange XL Site-directed Mutagenesis Kit (Stratagene). The wild-type sequence used in this study also harbors the common coding variant c.79A>C (p.Ile27Leu) (MAF = 34.8%) and a common synonymous variant c.51C>G (p.Leu17=) (MAF = 46.5%). All constructs were verified by DNA Sanger sequencing.

For transactivation experiments, two reporter constructs were used: (1) pGL3-RA, containing the promoter of the rat albumin gene (nucleotide −170 to +5) next to the Firefly *luciferase* gene in vector pGL3-Basic (Promega) (kindly provided by Prof. Graeme I. Bell, University of Chicago, Chicago, IL, USA) and (2) pGL3-HNF4AP2, which contains the human *HNF4A* (MIM: 600281) P2 promoter (nucleotide −418 to +13) next to the Firefly *luciferase* gene (kindly provided by Prof. Maria Angeles Navas, Madrid University, Madrid, Spain). The pRL-SV40 reporter vector containing the Renilla *luciferase* gene was used as internal control in the transactivation assay (Promega).

Transactivation Assays

Assessment of variant effects on transcriptional activity (TA) were performed in the HeLa cell line, representing cells negative for endogenous HNF-1A expression, and in the INS-1 (rat insulinoma cell line), representing cells positive for HNF-1A expression.

HeLa[20] and INS-1 cells[21] were grown as previously described. Transient transfection of variant plasmids (wild-type or variant *HNF1A*), reporter, and control plasmids was performed using Lipofectamine 2000 (Life Technology), as reported.[2] Luciferase activity was measured 24 h post-transfection with the Dual-Luciferase Assay System (Promega) in a Chameleon luminometer (Hidex) or using the Enspire platform (Perkin Elmer). Luciferase activity was normalized for transfection efficiency by the Renilla luciferase activity. The Bergen dataset included some variants previously reported (p.Ile27Leu, c.92G>A [p.Gly31Asp], c.142G>A [p.Glu48Lys], c.290C>T [p.Ala97Val], c.293C>T [p.Ala98Val], c.298C>A [p.Gln100Lys], c.341G>A [p.Arg114His], c.392G>A [p.Arg131Gln], c.965A>G [p.Tyr322Cys], c.1165T>G [p.Leu389Val], c.1405C>T [p.His469Tyr], c.1460G>A [p.Ser487Asn], c.1469T>C [p.Met490Thr], c.1541A>G [p.His514Arg], c.1544C>A [p.Thr515Lys], and c.1729C>G [p.His577Asp]).[2]

HNF-1A Protein Abundance

The level of wild-type and individual HNF-1A variant protein expressions in total HeLa cell lysates was determined. For this purpose, the team at Bergen used 20 μL of HeLa cell lysates generated for transactivation assays as previously described.[2] HNF-1A and actin protein levels were quantified by densitometric analysis using Quantity One 1-D software (Bio-Rad). The team at Oxford evaluated protein expression by transfecting HeLa cells with 5 μg of wild-type or variants plasmids after culturing for 24 h. Total protein quantification was carried out using Bradford reagent (Bio-Rad) and 10 μg of total protein was electrophoresed then immunoblotted with antibodies for HNF-1A (Santa Cruz Biotechnology) and beta-tubulin (Santa Cruz Biotechnology) and visualized using the ChemiDoc Imaging System (Bio-Rad). Densitometry (for western blots and EMSA) was carried out using Image Lab Software (Bio-Rad).

Subcellular Localization

For nuclear translocation assessments, the teams at Bergen and Oxford examined HNF-1A presence in nuclear versus cytosolic HeLa cell fractions. Cultured and plated cells were transiently transfected with wild-type or *HNF1A* variant plasmids. Sequential cell fractionation from each transfected sample was performed 24 h post-transfection as described.[22] 20 μg total protein from each isolated compartment (nucleus and cytosol) was analyzed by SDS-PAGE and immunoblotting using an HNF-1A-specific antibody (Cell Signaling or Santa Cruz Biotechnology, respective to the two centers). GAPDH antibody (Santa Cruz Biotechnology) and Topoisomerase II-alpha antibody (Cell) were used as loading control for cytosol and nuclear compartments, respectively. The *HNF1A* variant c.589_615del (p.Leu197_Leu205del), denoted p.delB, was included as a positive control for impaired nuclear localization (cytosolic retention).[23]

DNA Binding

DNA binding ability test was conducted for *HNF1A* variants that were located in DNA binding domain (1–287 aa), and those that demonstrated transactivation activity < 50%. At Bergen the HNF-1A proteins were expressed using an *in vitro* transcription and translation system (TNT Coupled Reticulocyte Lysate System, Promega) and equal amounts of synthesized protein were bound to a [γ-32P]-radiolabeled rat albumin oligonucleotide as described.[24] DNA-protein bound complexes were separated by 6% DNA retardation gel electrophoresis (EMSA) (Life Technologies) followed by autoradiography (LAS-1000 Plus, Fujifilm Medical System). Level of DNA binding was assessed by quantification of the intensity of HNF-1A protein-oligonucleotide complexes by the program Image Gauge 3.12 (Fujifilm Medical Systems). The two *HNF1A*-MODY control variants c.335C>T (p.Pro112Leu) and c.608G>A (p.Arg203His) were included as positive controls for reduced DNA binding ability.[24] DNA binding data for two variants p.Gln100Lys and p.Arg131Gln are the same as published previously.[2] The team at Oxford used 10 μg of the cell lysate used in the protein abundance western and a CY5 labeled probe of the same promoter sequence as used by the Bergen team. However, they used the Odessey Infra-Red EMSA kit (Li-Cor Inc.) to conduct binding affinity. The percentage of HNF-1A-oligo complexes for each variant fraction was then calculated compared to wild-type.

Stratification of *HNF1A* Variants with Unsupervised Learning Methods

We designed an analytical pipeline to stratify functionally characterized *HNF1A* variants along the spectrum of glycemic phenotypes (Figure S3). Briefly, the pipeline begins with preparation of the dataset for analysis using unsupervised learning tools. This was followed by the addition of two scores to each variant, one to account for functional domain and the other for spatial variation in *HNF1A* isoform expression (exon location), as there are well-established correlations between variant position in *HNF1A* and clinical phenotype.[13,14] The "polished" dataset was then processed using principal component analysis, k-means, and hierarchical clustering methods (Figure S3).The *prcomp (principal components analysis)* function in R (stats package v.3.5.0) was used to perform principal component analysis. Polished data matrices were zero centered and scaled to account for unit variance. We used the *NbClust* Package in R for determining the best number of clusters (distance measure set as "Euclidean") to estimate the optimal number of k-means clusters in PC space[25] (Figures S4A and S4B). Hierarchical clustering was performed on a Euclidean distance matrix comprised of PC scores for each allele from total principal components that explained >85% of data variance (Figures S4C and S4D) using the *hclust (hierarchical clustering)* function in R. The WARD minimum variance hierarchical clustering method (ward.d2) was selected as it yielded highest resolution clusters in a comparative analysis against complete, single, and average linkage methods (data not shown) based on (1) predicted grouping patterns of wild-type and MODY/type 2 diabetes risk reference variants and (2) known molecular function of variants which co-occupied clusters defined by wild-type, type 2 diabetes risk, and MODY reference variants. To compare the cluster dendrograms of variants shared between Oxford and Bergen, we used the untangle, tanglegram, and entanglement functions in R (part of the dendextend package) to untangle dendrogram lists and find the best alignment layout, plot the two dendrograms side by side, and compute the quality of alignment (entanglement coefficient), respectively.

Dataset Preparation for Clustering Analysis

The functional datasets were prepared for PCA and clustering analysis by harmonizing the number of variables across tested variants. DNA binding ability was interrogated for only a small subset of variants, so EMSA data were excluded. Functional data were available in three different formats: raw instrument data, data normalized to internal assay controls (renilla luciferase for TA assay, beta-tubulin/actin antibody for protein abundance

assay, and nuclear:cytosolic ratio of raw protein abundance reads for nuclear localization) expressed as biological replicates, and fully processed summary data normalized to wild-type values. The most statistically suitable input format for PCA and unsupervised clustering is functional data normalized to internal assay controls (semi-processed) as intra-assay measurements are harmonized (versus raw instrument data) and the organic structure of the data is retained and uninfluenced by assumptions (versus wild-type normalized data). Further, this format yielded the most robust clustering trends based on distribution quality in multivariate space and known and expected sequence-function relationships. Scores for tissue-specific expression of *HNF1A* isoforms (implications for clinical phenotypic manifestation) and functional domain (varied levels of mutation tolerance) were assigned to each variant (Table S3). For stratification of *HNF1A* variants with unsupervised learning methods, see Figure S4 and Material and Methods.

Variant-Phenotype Mapping

We surveyed the UK MODY Diagnostic Registry (Royal Devon and Exeter NHS Foundation Trust, Exeter, UK) and the Norwegian MODY Registry (Haukeland University Hospital, Bergen, Norway) for functionally annotated *HNF1A* missense variants. A total of 162 and 53 *HNF1A* missense variants were documented in the UK and Norwegian diagnostic registries, respectively. Tables S4 and S5 show the list of clinical features that were available from the database for alleles which overlapped with the Oxford-Bergen dataset (not all features available for each variant). Sequence variants in the Norwegian MODY Registry were classified prior to the incorporation of the ACMG/AMP guidelines,[12] as described[26] using a 5-tier score system.[27] Sequence variants in the Exeter MODY Registry had been classified using the ACGS guidelines from 2013 (see Web Resources), a 5-tier system used in the UK prior to the advent of the ExAC database and publication of the ACMG/AMP guidelines.[12] Original clinical reports of carriers were accessed for additional details, particularly where clinical features were sparse—such as extra-pancreatic features, vascular complications, additional family history data, and whether, for example, other MODY genes were next-generation sequenced as part of a MODY gene panel.[28] The classification system adopted by each center was used for reclassification of variants from its database.

Role of the Funding Source

The funders of the study had no role in study design, data collection, data analysis, data interpretation, or writing of the report. The corresponding author had full access to all the data in the study and had final responsibility for the decision to submit for publication.

Results

In Silico and *In Vitro* Functional Characterization of Variants

To resolve *HNF1A* genotype-phenotype complexity, we sought to evaluate the function of 73 *HNF1A* missense alleles which were observed almost 26K times in the exomes of ~13K multi-ethnic type 2 diabetes case subjects and control subjects. The majority of *HNF1A* variants were identified in both type 2 diabetes case subjects and control subjects, and for the few observed exclusively in type 2 diabetes case subjects, they were identified with a frequency of either one or two case subjects per variant (Table S1). Although there was no evidence for *HNF1A* association with type 2 diabetes susceptibility in the study either at the single variant or at the gene level, there was a marginal aggregate association with type 2 diabetes risk in a multigene test ($p = 0.023$) which included rare coding alleles in a set of genes implicated in monogenic/syndromic diabetes or related glycemic traits.[15] Consensus across multiple *in silico* tools for predicting pathogenicity was observed in only 38 of the 73 variants (53%) (Table S1). Based on CADD scores (suggested pathogenicity cut-off > 15), ~70% of the missense variants would be bioinformatically classified as disease causing (i.e., sufficient to cause MODY).[19]

The 73 *HNF1A* variants were divided between the two centers (Oxford and Bergen) and individually evaluated in terms of functional effect using a common pipeline including assays measuring variant effect on HNF-1A transcriptional activity, subcellular localization, protein expression level, and DNA binding ability (Figures S1–S3). The Oxford laboratory investigated variants predominantly of South-East Asian etiology, while the Bergen laboratory studied variants mainly of European etiology.

The variants demonstrated a wide range of functional effects from benign to damaging across assays and laboratories with highest variability in transactivation assessments, particularly through regulation of the rat albumin promoter in HeLa cells (activity range 30%–110% in Bergen data [Figure S5A], 52%–114% in Oxford data [Figure S6A]). Transcriptional activity was consistently higher for variants using *HNF4A* P2 promoter in INS-1 cells (versus rat albumin promoter in HeLa cells) (activity range 40%–90% Bergen data, 77%–158% Oxford data), and most likely due to interference of endogenous HNF-1A in INS-1 cells (2- to 4-fold higher basal promoter activity, Figures S5B and S6B). In assessments of protein abundance, >85% of all variants displayed adequate HNF-1A protein levels (>60%, Figures S5C and S6C). Similarly, in nuclear translocation assays, most variants were predominantly detected in the nucleus (level > 60%), the exception being five variants from the Bergen dataset (c.827C>A [p.Ala276Asp], p.Ser487Asn, c.1812C>G [p.Ser604Arg], c.1322C>A [p.Thr441Lys], p.Arg131Gln) (Figures S5D and S6D) from the Oxford dataset (c.185A>G [p.Asn62Ser], c.1552C>T [p.Leu518Phe], c.1605C>A [p.Ser535Arg], c.1610C>T [p.Thr537Met], c.1748C>A [p.Arg583Gln]) displaying < 50% level. The subsets of variants investigated by EMSA demonstrated overall normal DNA binding ability (~95% of variants > 60%), with the exception of one variant from the Bergen dataset (p.Arg131Gln < 50%) and five from Oxford dataset (p.Asn62Ser, c.340C>T [p.Arg114Cys], c.467C>T [p.Thr156Met], c.481G>A [p.Ala161Thr], p.Ser535Arg < 50%) (Figures S5E and S6E).

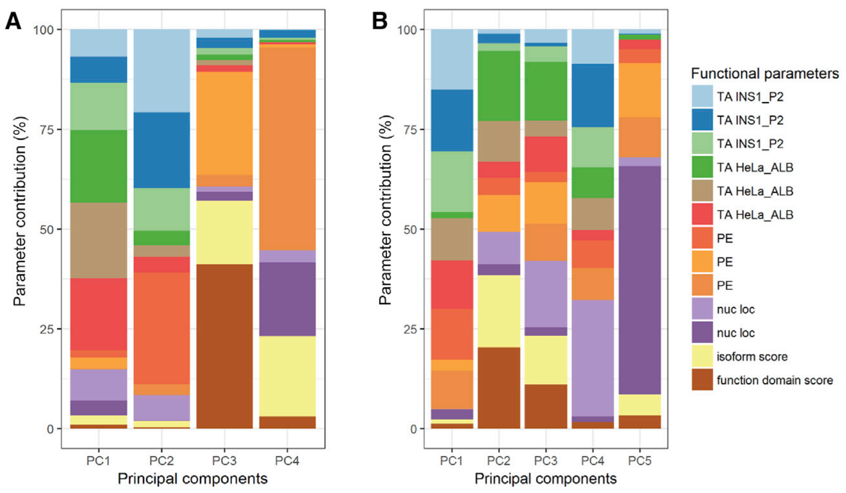

Figure 1. Eigendecomposition of Principal Components Explaining > 85% of Variance
Shown are (A) Oxford and (B) Bergen datasets. TA INS1_P2 and TA HeLa_ALB are transcriptional activity data from INS-1 cells using *HNF4A* P2 promoter and from HeLa cells using rat albumin promoter, respectively. PE, protein expression; nuc loc, nuclear localization data.

Multi-Dimensional Data Analysis

We performed unsupervised stratification of the *HNF1A* variants using the multi-dimensional *in vitro* functional data (semi-processed data normalized to internal technical controls in each assay) supplemented with scores for isoform expression and functional domain, with the aim of mapping molecular dysfunction to clinical phenotype. Each of the two datasets were analyzed independently to minimize the interference of inter-laboratory variability with true biological signal. We used principal component analysis to facilitate informative dissection and visualization of multi-parametric functional data. Eigendecomposition of the data matrices revealed transcriptional activity as the greatest contributor to data variance and structure (Figure 1). To enable variant subgroup discovery for function-phenotype mapping, data were partitioned using (1) k-means clustering in PC space (Figure 2) and (2) hierarchical clustering using data coordinates from the total number of informative principal components for each dataset (Figure 3). The analysis yielded variant clusters neatly organized along the spectrum of *HNF1A* molecular dysfunction ranging from neutral/benign to intermediate to damaging. As such, we broadly annotated the known *HNF1A in vivo* spectrum, from benign to type 2 diabetes risk-modifying to HNF1A-MODY (inherited early-onset hyperglycemia, likely to be sulfonylurea-responsive based on MODY registry data), onto the principal components plots and dendrograms based on the spatial distribution of the variants along the *in vitro* data-derived functional spectrum, from wild-type/wild-type-like to intermediate to damaging (Figures 2 and 3). To understand (mis)alignment of *HNF1A* variants shared between the two centers, we visually compared the cluster dendrograms of shared variants only (Figure S7). We computed an entanglement coefficient (the quality of the alignment of the two dendrograms expressed as a value from 0 to 1 where lower values correspond to higher quality alignment) of 0.055 which indicated good high-quality alignment of shared variants (Figure S7).

Clinical Interpretation of *HNF1A* Variants

To assess the medical diagnostic utility of multi-tiered *HNF1A* sequence-function annotations, we examined their mapability to *HNF1A* clinical phenotype using clinical data from overlapping *HNF1A* missense variants in the UK and Norway MODY diagnostic registries (Tables S4 and S5).

Of the 31 total overlapping variants between our functional effort and the UK registry, 19 were originally classified as pathogenic/likely pathogenic and 15 as VUS/likely benign in the diagnostic registry. Three of the 31 overlapping variants (c.1816G>A [p.Gly606Ser], p.His469Tyr, c.871C>T [p.Pro291Ser]) were present under both pathogenic/likely pathogenic (where they were considered the MODY-causal variant in the case subjects) and VUS/likely benign (cases of co-occurrence with a pathogenic variant in *HNF1A* or another MODY gene) original clinical classifications. All 15 missense variants categorized as VUS/likely benign in the UK database demonstrated benign clustering patterns in our analysis (i.e., did not form subgroups with variants which exhibited impaired function). However, for 10 of the 19 variants clinically categorized as pathogenic/likely pathogenic in the UK diagnostic database (p.Ala161Thr, c.521C>T [p.Ala174Val], c.139G>C [p.Gly47Arg], p.Gly606Ser, p.His469Tyr, c.1235T>C [p.Met412Thr], p.Asn62Ser, p.Pro291Ser, p.Arg131Gln, c.29C>T [p.Thr10Met]), patterns of *in vitro* functional clustering patterns did not match clinical diagnostic variant interpretation. The variants co-occupied clusters either with known type 2 diabetes risk modifiers (some moderately impacted in functional assays) or with wild-type/neutral variants. Discordance between functional genotype and clinical variant interpretation prompted a thorough reassessment of variant pathogenicity.

The missense variant p.Asn62Ser (gnomAD allele count n = 33) was consistently dissimilar to dysfunctional variants in dendrograms and k-means derived clusters from both Bergen and Oxford datasets (Figures 2 and 3). In the UK MODY registry, it was identified in an obese individual who was diagnosed with diabetes at age 36 years (Table S6). The patient suffered from microvascular complications (MIM: 603933) (nephropathy and retinopathy) (Table S6). These features are inconsistent with neither HNF1A-MODY nor a type 2 diabetes phenotype (which might be

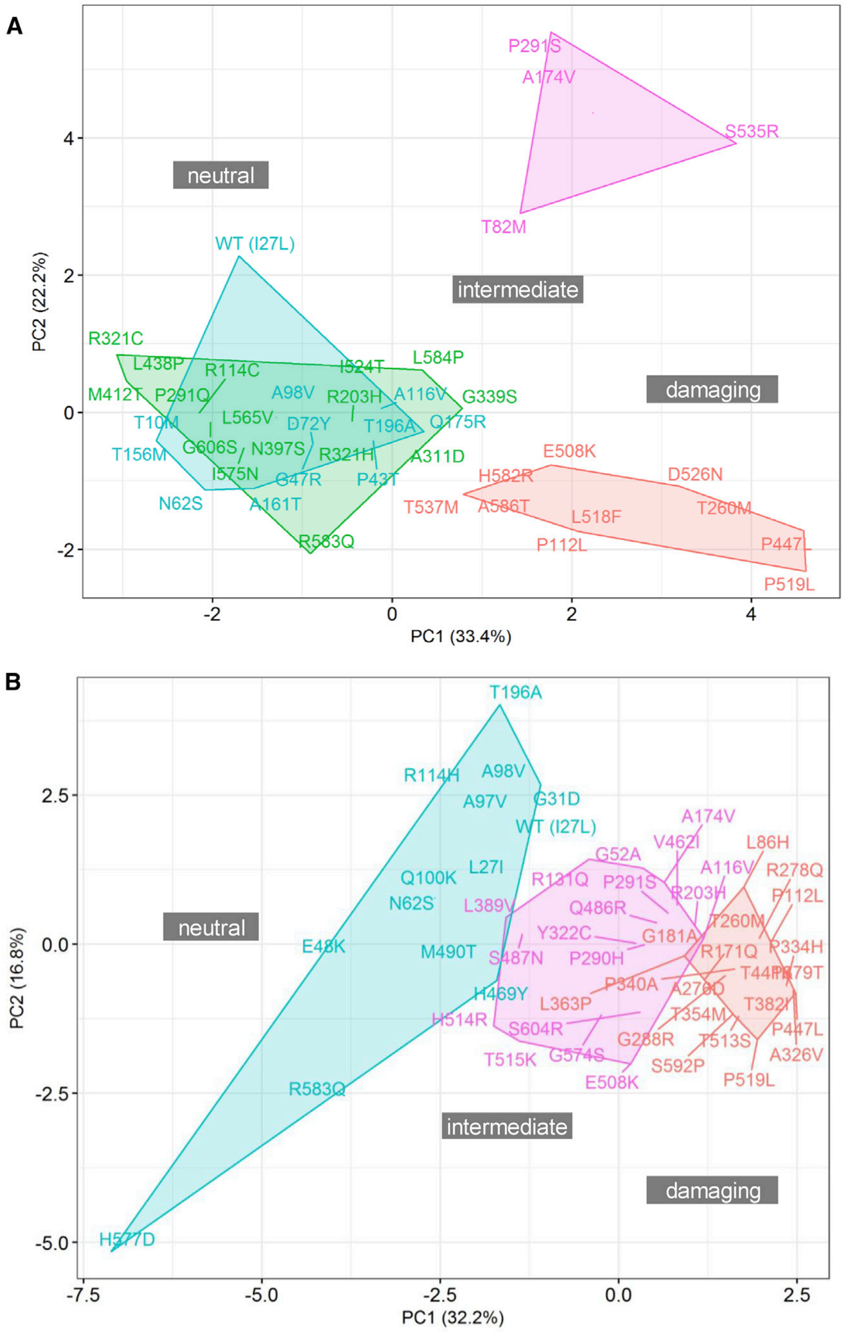

Figure 2. K-Means Clustering
HNF1A missense alleles characterized at Oxford (A) and Bergen (B) in principal component (PC) space. Blue and green k clusters represent alleles with benign and benign-to-intermediate effects on function, respectively; purple k clusters represent alleles with intermediate functional impact; red k clusters indicate intermediate-to-damaging or functionally damaging alleles.

familial considering the number of affected individuals in the carrier's pedigree). *HNF1A* was the only MODY gene sequenced in this individual as genetic testing was performed before the advent of the targeted MODY gene exome sequencing panel which is the current diagnostic procedure. Alone, p.Asn62Ser allele frequency values are sufficient to confidently re-categorize the variant as VUS/likely benign in the context of MODY (Figure 4).

The variants p.Ala174Val and p.Pro291Ser, characterized by both laboratories, were more difficult to interpret. In the Oxford dataset, these variants formed a separate outlying k-means-derived cluster (Figure 2). They also occupied an independent subgroup in hierarchical clustering which branches high on the height scale away from the larger cluster defined by wild-type and other neutral variants (Figure 3). Atypically high luciferase renilla values (internal luciferase reporter gene assay control used for normalization) were reported for these variants as well as for p.Ser535Arg, which have resulted in a potentially exaggerated reduction in transactivation values for these variants upon normalization to the internal assay reference in the Oxford dataset (Figure S6). In the Bergen dataset, these variants also consistently lie in the type 2 diabetes risk modifier zone (not pathogenic for MODY) (Figures S2 and S4). Not only are the activity profiles of p.Ala174Val and p.Pro291Ser dissimilar to those of pathogenic MODY variants, they also occur at a much higher frequency in the general population (Figure 4). In terms of clinical profiles, p.Ala174Val was detected in an individual with diet-controlled diabetes, which was diagnosed at age 25 years (Table S6). The p.Pro291Ser variant was detected in an overweight individual who was diagnosed with diabetes at age 42 years when it was classified as likely pathogenic/pathogenic (Table S6). In another unrelated individual, p.Pro291Ser was co-expressed with p.Gly31Asp and both were annotated as VUS/likely benign in the diagnostic database.

The clinical diagnostic classifications of p.Gly606Ser and p.Ala161Thr did not match clustering patterns in multivariate space (Figures 2 and 3). The variants did not impact HNF-1A function in the *in vitro* assays tested. The frequency associated with these variants (n = 12 alleles in gnomAD and n = 2 in the Exeter diagnostic clinic) are inconsistent with those of rare MODY-causing variants. The p.Gly606Ser variant has also been found in a single case of hyperinsulinemic hypoglycemia (on diazoxide treatment; MIM: 256450) in the UK registry and in this case was classified as VUS/likely benign.

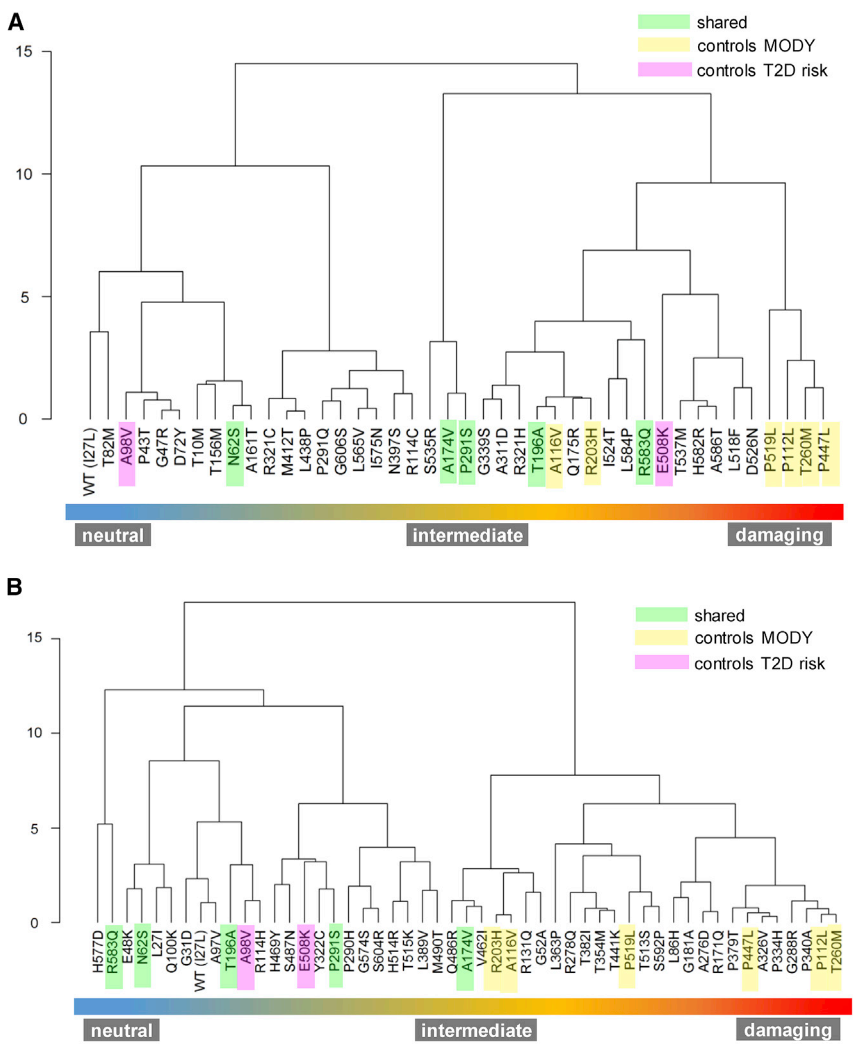

Figure 3. Hierarchical Clustering Analysis
HNF1A missense alleles characterized at Oxford (A) and Bergen (B). WARD minimum variance method was used and analysis performed using orthogonally transformed functional data from PC1-PC4 (>85% explained variance) from Oxford dataset and PC1-PC5 (>85% explained variance) form Bergen dataset. To optimize visualization of the function phenotype gradient, some branches were rotated. The numbers of the y axes of (A) and (B) refer to clustering height calculated as by Ward's criterion (total within-cluster variance).

In clustering analysis, p.His469Tyr occupied either the same or highly similar (adjacent) subgroups as wild-type (Leu27 and Ile27) (Figure 3). The clinical features of the variant carrier described in Table S6 are consistent with severe young-onset familial diabetes; however, the allele high frequency in gnomAD is 32. Another variant in *HNF1A*, c.620G>A (p.Gly207Asp) (not present in gnomAD), was, however, detected in the same individual. It was identified in three other case subjects (including co-occurrence with p.His469Tyr) and was classified as pathogenic/likely pathogenic each time it was identified in the UK MODY Registry. Thus, it is likely that p.Gly207Asp is the MODY-causal variant and that p.His469Tyr is either benign or potentially type 2 diabetes risk-modifying.

Despite alignment between clustering pattern and clinical diagnostic interpretation (Figures 2 and 3), c.1576G>A (p.Asp526Asn) was reclassified from pathogenic/likely pathogenic to VUS/likely benign. In transactivation assays, HNF1A-p.Asp526Asn was the most impaired of all tested exome-identified variants in the Oxford dataset (~50% in HeLa and ~80% in INS-1 cells; MODY reference variants exhibited transactivation range of 20%–40% in HeLa cells and 30%–50% in INS-1 cells in Oxford, with the exception of p.Arg203His and c.347C>T [p.Ala116Val], which yielded transactivation values of 40% and ~60% in HeLa and ~50 and ~100% in INS-1 assays, respectively). The variant is observed only five times in gnomAD, which suggests it might not be causal for MODY (Figure 4). The clinical profile of the p.Asp526Asn carrier did not appear to be consistent with HNF1A-MODY, besides presence of diabetes in three generations of the carrier's family. The variant carrier had BMI 32.4 kg/m^2 and was diagnosed with diabetes at age 33 years. Other clinical features included dyslipidemia (MIM: 144250), polycystic ovary syndrome (MIM: 184700), insulin resistance (MIM: 610549), and hypertension (MIM: 145500). The variant was also found in a patient in the Norwegian MODY registry. This patient, diagnosed at 19 years of age, had normal BMI and C-peptide levels. Type 1 diabetes (MIM: 222100) autoantibody status and type 1 diabetes risk score were not known. The carrier was treated with metformin. His mother and the mother's brother also have diabetes (treated with diet and insulin, respectively). Moreover, the patient was diagnosed with Crohn disease (MIM: 266600). Altogether, this suggests that the carriers might have a combination of type 2 diabetes and HNF1A-MODY, which is not uncommon, or a phenotype representing a possible continuum of diabetes sub-phenotypes from MODY to type 2 diabetes.[5] Further, the variant is expressed in the hepatocyte-dominant isoform and is thus unlikely to manifest in a strong beta-cell phenotype despite its poor functionality.

Of the 19 *HNF1A* missense variants that overlapped with the Norwegian MODY Registry, 18 were originally classified as benign (class 1), likely benign (class 2), or VUS (class 3), and 1 (p.Ala276Asp) as likely pathogenic (class 4) in the diagnostic registry. The variant p.Ala276Asp consistently demonstrated impaired HNF-1A function in *in vitro* assays

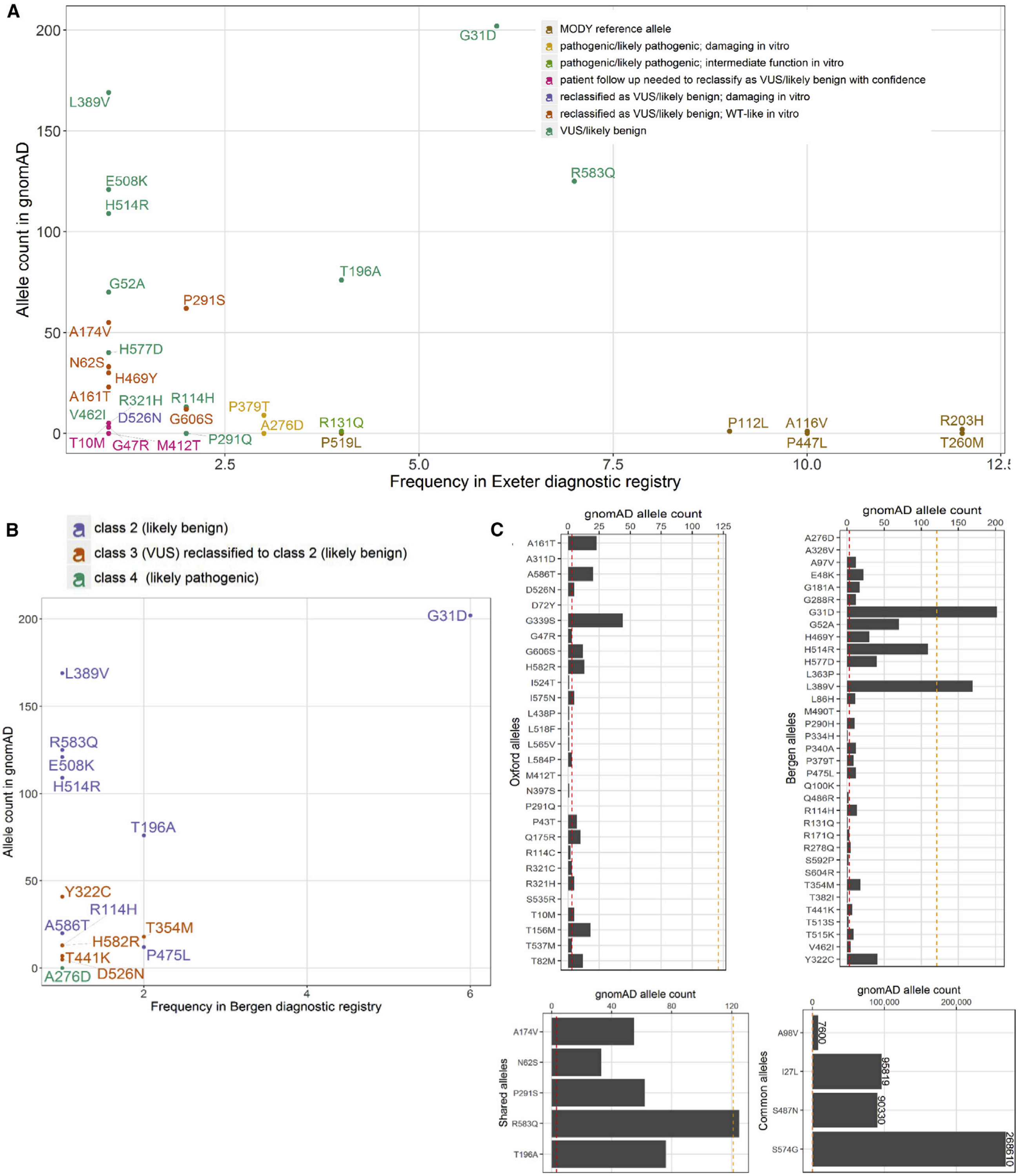

Figure 4. Distribution of Functionally Annotated *HNF1A* Missense Alleles
(A and B) As a function of frequency in the (A) UK MODY diagnostic registry and (B) Norway MODY diagnostic registry on the x axis and reported frequency in the genome aggregation database (gnomAD) on the y axis. Alleles are colored on the basis of the (re)classification scheme on the top right.
(C) Frequency of functionally characterized exome-detected *HNF1A* missense alleles in gnomAD. The red and orange dashed lines mark known ultra-rare, MODY pathogenic (allele count ≤ 2, AF $< 0.0008\%$) and low frequency type 2 diabetes predisposing allele frequencies (allele count ≤ 121, AF $< 0.04\%$) respectively.

and clustered with the MODY reference variants in the unsupervised clustering analyses, supporting the clinical interpretation of this variant as pathogenic (Figures 2 and 3**)**. It was also clinically classified as likely pathogenic/pathogenic in the UK MODY registry (Figure 4). All variants originally classified as benign/likely benign/VUS (class 1–3) in the Norwegian registry clustered in the benign or intermediate type 2 diabetes risk modifier zones, with the exception of four variants (c.1016C>T [p.Thr354Met], p.Thr441Lys, c.1745A>G [p.His582Arg], c.1756G>A [p.Ala586Thr]), which demonstrated variable trends across clustering methods (Figures 2 and 3).

In k-means clustering along principal component 1 and principal component 2, these variants co-occupy a hard cluster with MODY reference variants and variants which exhibited damaging *in vitro* function. This is not entirely unexpected for HNF1A-p.Thr441Lys which displayed reduced activity (~50% on both promoters in INS-1 and HeLa cells) and with reduced (<40%) nuclear localization. Although, in hierarchical clustering, where (dis)similarity between variants was determined using principal component scores from all principal components contributing to >85% of overall variance, the trends were more consistent with clinical features and classification; p.Thr441Lys and p.Thr354Met are in the type 2 diabetes risk modifier space of the *in vivo* continuum, hierarchically distanced from the sub-cluster defined by the majority of MODY reference variants and pathogenic damaging variants p.Ala276Asp and c.1135C>A (p.Pro379Thr). The clinical phenotypic data of the p.Thr354Met variant carriers seems more consistent with type 1 diabetes. The p.Thr354Met variant was identified in two unrelated individuals. Upon revisiting clinical data on these two allele carriers, it was found that one of the carriers with insulin-treated diabetes from age 14 years was positive for GAD and IA2 autoantibodies. A sister of the proband had diabetes, but the parents were apparently unaffected. The other p.Thr354Met variant carrier had autoantibody-negative diabetes from age 12 years without strong family history of diabetes (grandmother only). Moreover, the population frequency (n = 18 in gnomAD) associated with this variant allele is inconsistent with rare, causal MODY alleles. Thus, the clinical phenotypic data of the p.Thr354Met variant carriers seem more consistent with type 1 diabetes. In the p.Thr441Lys variant carrier, another variant in *HNF1A* c.872dup (p.Gly292Argfs*25) was considered the pathogenic MODY variant (Table S7). Moreover, the population frequency values of p.Thr441Lys (gnomAD allele count n = 18) and p.Thr354Met (gnomAD allele count n = 7) are slightly higher than expected for rare disease-causing variants (Figure 4). As for p.His582Arg (gnomAD allele count n = 14) and p.Ala586Thr (gnomAD allele count n = 20), in hierarchical clustering, these variants form a subgroup defined by liver isoform variants which demonstrated suboptimal function in one or more *in vitro* assays. Much like p.Asp526Asn, these variants are likely to be strong type 2 diabetes risk modifiers. The p.His582Arg variant carrier was diagnosed with diabetes age 11 years, she had a BMI of 29 at referral one year later, and C-peptide was measured to 1,000 pmol/L (Table S7). The p.Ala586Thr variant carrier was diagnosed at age 11, C-peptide positive (78 pmol/L), negative GADA, IA2A, ZnT8A, with no known family history of diabetes, and treated with insulin (Table S7).

Based on this comprehensive variant re-assessment effort, we changed the classification of 7 out of 31 variants shared with the UK MODY diagnostic database (p.Ala161Thr, p.Ala174Val, p.Gly606Ser, p.His469Tyr, p.Asn62Ser, Pro291Ser, p.Asp526Asn) from likely pathogenic to VUS/likely benign (Figure 4) and all five variants categorized as VUS (class 3) or VUS/likely benign (class 3−) in the Norway MODY registry (p.Tyr322Cys, p.Thr354Met, p.Thr441Lys, p.Asp526Asn, and p.His582Arg) to likely benign (class 2) (Figure 4). This represents ~23% and ~26% of total *HNF1A* missense variants in the UK and Norway MODY registries, respectively, that overlap with the functionally interrogated *HNF1A* missense variants detected in the exomes of ~13K multiethnic type 2 diabetes cases and controls.

Discussion

In this study, we investigated the functional impact of 73 missense variants in *HNF1A*, detected by exome sequencing of a multi-ethnic type 2 diabetes case-control cohort from four different mechanistic angles (Figures S1 and S3). We developed an approach for the analysis of multi-parametric functional data which, in the context of *HNF1A,* has enabled (1) a holistic assessment of variant behavior by combining as many mechanistic dimensions as possible, (2) unbiased stratification along the spectrum of glycemic phenotypes ranging from neutral/benign effects, to modification of multifactorial polygenic diabetes risk, to deleterious and causal for early-onset sulfonylurea-responsive diabetes, (3) an assessment of the relative contributions of each functional parameter to molecular variability, and (4) rigorous phenotype mapping and a thorough re-evaluation of the clinical classifications of overlapping variants in two national MODY diagnostic registries.

Revisiting clinical variant classifications using *HNF1A* functional clusters led to the reclassification of ~4% (7/162) and ~9% (5/53) of all *HNF1A* missense variants in the UK and Norwegian MODY diagnostic registries, respectively. Decisions on variant reclassification were primarily motivated by the juxtaposition of allele frequency values in the general population (based on gnomAD allele counts) against their frequency in the MODY diagnostic registries (highest frequency values belonged to bona fide loss-of-function alleles used as MODY reference controls in this study) (Figure 4). This is based on the rules given by the ACMG guidelines for variant interpretation: "*an allele frequency in a control population that is greater than expected for the disorder is considered strong support for a benign interpretation for a rare Mendelian disorder*" (BS1).[12] Information

from other layers of variant annotation such as *in vitro* function (in the tested assays), clinical features, family history, ethnicity, and *in silico* prediction all helped to support reclassification decisions.

Dissection of the individual principal components revealed transactivation to be the primary contributor to the spatial distribution of multi-parametric data. This suggests that it might be a superior functional readout and potentially more informative than other molecular assays for assessing *HNF1A* variant pathogenicity, and in line with our previous experience on various functional assays of *HNF1A* variants.[2] Since transactivation is a relatively all-encompassing measure of transcription factor protein function, we assumed defects in transcriptional activity would capture defects in its biochemical prerequisites (protein expression, nuclear transport, DNA binding). While this may have been the case for the majority of functionally interrogated *HNF1A* variants, p.Arg203His and p.Ala116Val highlight the limitations of this assumption. For these variants, severely impaired DNA binding ability was not adequately captured by transactivation. This might explain clustering of p.Ala116Val and p.Arg203His in the intermediate zones among type 2 diabetes risk modifiers, and not directly among MODY reference alleles, in both k-means and hierarchical clustering for both centers (Figure 3).

We were able to mitigate error associated with handling data from two centers with methodological differences by benchmarking several HNF-1A variants (benign, type 2 diabetes risk, and MODY) in both laboratories. Discrepancies between the two centers with respect to shared variants can be explained in part by variability in technical protocols between laboratories and the handling of samples by various individuals over the course of the study. The relative clustering position of the shared variants is impacted by the function trends observed in each dataset; while the majority of Oxford variants behaved wild-type-like, the Bergen dataset was more complex as variants demonstrated a wider range of effects. For instance, for an intermediate variant and a known type 2 diabetes risk modifier such as p.Glu508Lys, the dissimilarity to MODY reference alleles is more pronounced in the Bergen dataset where there are more data points between moderately impaired and damaging function (Figure S7).

While important, informative, and powerful first lines of evidence, functional annotations should not be treated as superior or stand-alone determinants of variant pathogenicity, which they have not in this study. Indeed, the same variant in a MODY gene can give rise to a spectrum of clinical phenotypes and exhibit variable penetrance depending on genomic (regulatory variants in *cis* or *trans*, or haplotype epistasis) and environmental (epigenomic) context which are difficult to capture in functional assessments.[29–33] It is also entirely possible that some of the noise in functional-clinical mapping is a reflection of the heterogeneity in the clinical phenotypic manifestation of *HNF1A* variants.[5] The same variant which has a mild effect on *HNF1A* function, and thus beta-cell function and ability to respond appropriately to a given level of glycemia, could play out differently in individuals who are already struggling to meet the insulin demand through insulin resistance and/or other genetically driven defects in their beta-cells.[34] Another aspect to consider is the expected variation in clinical practice between the two centers in the UK and Norway to which diabetes patients have been referred. It would thus be naive to attempt to draw conclusions regarding variant effects *in vivo* from, for example, a single registry observation. The reality of phenotypic variant manifestation is often complex, context dependent, nonlinear, and spectrum based. Developing a contextual and thorough understanding of variant behavior from diverse functional, clinical, biochemical, and demographic datasets is necessary to facilitate highly accurate interpretation.

The p.Asp526Asn variant in *HNF1A* is a perfect example that illustrates the need for nuanced evaluations of variant effects despite the availability of multiple layers of functional annotation from various cell systems. The variant was clinically classified as pathogenic/likely pathogenic and exhibited impaired *in vitro* functional activity (shared a cluster with known MODY-causal variants). Its impact on molecular function would be consistent with biomarker profiles (hsCRP and glycans) suggestive of HNF1A-MODY. Yet, re-evaluation of the clinical features of variant carriers in UK and Norway diabetes registries and the fact that it is present only in the longest *HNF1A* transcript isoform expressed predominantly in liver suggest that it is more likely to be a contributing factor to common multifactorial diabetes rather than a primary driver of early-onset sulfonylurea-responsive familial hyperglycemia. Integration of isoform weights into the unsupervised clustering model helped separate bona fide loss-of-function MODY variants from functional variants expressed in exons 8–10 at the lowest level of hierarchical clustering.

The recent advent of multiplexed assays of variant effects (MAVEs) has made it possible to interrogate the function of every possible sequence perturbation in a single experimental system.[35] Successful and productive implementation of these technologies requires overcoming the technical and analytical complexities associated with scaling up, which represent the most significant barrier in the face of closing the chasm between variant resolution and variant interpretation. A meticulously designed MAVE for *HNF1A* would enable functional annotation of all possible missense variants (>12,000) in a single assay. A high-performance variant classifier built using MAVE-based data can then be used to generate an exhaustive catalog of variant effects which researchers and clinicians can consult upon sequence-identification of an *HNF1A* variant.

The performance and predictive utility of any model built using *HNF1A* MAVE-derived function scores would be enhanced immeasurably upon calibration against these multi-layered data and comprehensively annotated function-clusters. These data can also improve existing

prediction algorithms which operate on the basis of multifactorial probability and multi-data integration such as CADD (Combined Annotation-Dependent Depletion), MutationTaster, FitCons (fitness consequence), and VAAST (Variant Annotation, Analysis and Search Tool). Further, they can be incorporated into rigorous and collaborative multi-level annotation efforts led by the Clinical Genome Resource (ClinGen) program and evidence-based disease-specific variant classification databases. Lastly, our approach can assist in filtering *HNF1A* missense variants for gene burden testing of rare variants. An immediate example is its application to the ~50K exomes in the UK Biobank not ascertained on the basis of diabetes. At present, in the UK Biobank dataset, the number of carriers of *HNF1A* variants that overlap with variants functionally investigated in our effort are insufficient to conduct a robust gene burden analysis. However, we have observed that seven variants present in the UK biobank and predicted by our functional data to be likely damaging (p.Asp526Asn, p.Arg171Gln, p.Thr354Met, p.Gly288Arg, p.His582Arg, p.Pro379Thr, p.Ser592Pro) were not identified in patients with diabetes; it is possible that these alleles represent very rare pathogenic variants causing MODY with reduced penetrance or are type 2 diabetes risk variants.

In conclusion, we have developed an analytical framework for robust and unbiased variant stratification using multi-dimensional functional follow-up data from the largest number of exome-identified missense variants in *HNF1A* ever studied. This allowed us to annotate functional clusters with clinical knowledge and identify discordant classifications between functional genotype and clinical phenotype. We believe our pipeline is an important proof-of-principle technical contribution on the path toward more reliable, scalable, and comprehensive mapping of sequence-function relationships: a significant factor in making well-informed initial judgements of allele pathogenicity in the context of individual phenotypic presentations.

Data and Code Availability

Additional data and code from this study is available upon reasonable request from the corresponding author.

Supplemental Data

Supplemental Data can be found online at https://doi.org/10.1016/j.ajhg.2020.08.016.

Acknowledgments

A.L.G. is a Wellcome Senior Fellow in Basic Biomedical Science. S.E. and M.I.M. are Wellcome Senior Investigators. This work was funded in Oxford by the Wellcome (095101 [A.L.G.], 200837 [A.L.G.], 098381 [M.I.M.], 106130 [A.L.G., M.I.M.], 203141 [A.L.G., M.I.M.], 203141 [M.I.M.]), Medical Research Council (MR/L020149/1 [M.I.M., A.L.G.]), European Union Horizon 2020 Programme (T2D Systems [A.L.G.]), and NIH (U01-DK105535; U01-DK085545 [M.I.M., A.L.G.]). The research was funded by the National Institute for Health Research (NIHR) Oxford Biomedical Research Centre (BRC, [A.L.G., M.I.M.]). This work was funded in Bergen by grants from the European Research Council (#293574 [P.R.N.]), the Research Council of Norway (#240413/F20 [P.R.N.]), Stiftelsen Kristian Gerhard Jebsen (P.R.N.), the Novo Nordisk Fonden (#54741 [P.R.N.]), the Western Norway Health Authorities (#911745 [P.R.N.]), and the University of Bergen (I.A., L.A.N., P.R.N.).

Declaration of Interests

The views expressed in this article are those of the authors and not necessarily those of the NHS, the NIHR, or the Department of Health. M.I.M. has served on advisory panels for Pfizer, NovoNordisk, and Zoe Global, received honoraria from Merck, Pfizer, NovoNordisk, and Eli Lilly, and received research funding from Abbvie, Astra Zeneca, Boehringer Ingelheim, Eli Lilly, Janssen, Merck, NovoNordisk, Pfizer, Roche, Sanofi Aventis, Servier, and Takeda. As of June 2019, M.I.M. is an employee of Genentech and a holder of Roche stock. A.L.G. reports grants from Wellcome Trust, grants from NIHR Oxford Biomedical Research Centre, grants from Horizon 2020, grants from NIDDK, and grants from MRC during the conduct of the study and personal fees from NovoNordisk and Merck outside the submitted work.

Received: January 9, 2020
Accepted: August 14, 2020
Published: September 9, 2020

Web Resources

ACGS Guidelines, https://www.acgs.uk.com/quality/best-practice-guidelines/
OMIM, https://www.omim.org
UK Biobank, https://www.ukbiobank.ac.uk/

References

1. Murphy, R., Ellard, S., and Hattersley, A.T. (2008). Clinical implications of a molecular genetic classification of monogenic beta-cell diabetes. Nat. Clin. Pract. Endocrinol. Metab. *4*, 200–213.
2. Najmi, L.A., Aukrust, I., Flannick, J., Molnes, J., Burtt, N., Molven, A., Groop, L., Altshuler, D., Johansson, S., Bjørkhaug, L., and Njølstad, P.R. (2017). Functional investigations of HNF1A identify rare variants as risk factors for Type 2 diabetes in the general population. Diabetes *66*, 335–346.
3. Estrada, K., Aukrust, I., Bjørkhaug, L., Burtt, N.P., Mercader, J.M., García-Ortiz, H., Huerta-Chagoya, A., Moreno-Macías, H., Walford, G., Flannick, J., et al.; SIGMA Type 2 Diabetes Consortium (2014). Association of a low-frequency variant in HNF1A with type 2 diabetes in a Latino population. JAMA *311*, 2305–2314.
4. Balamurugan, K., Bjørkhaug, L., Mahajan, S., Kanthimathi, S., Njølstad, P.R., Srinivasan, N., Mohan, V., and Radha, V. (2016). Structure-function studies of HNF1A (MODY3) gene mutations in South Indian patients with monogenic diabetes. Clin. Genet. *90*, 486–495.

5. Flannick, J., and Florez, J.C. (2016). Type 2 diabetes: genetic data sharing to advance complex disease research. Nat. Rev. Genet. *17*, 535–549.
6. Flannick, J., Beer, N.L., Bick, A.G., Agarwala, V., Molnes, J., Gupta, N., Burtt, N.P., Florez, J.C., Meigs, J.B., Taylor, H., et al. (2013). Assessing the phenotypic effects in the general population of rare variants in genes for a dominant Mendelian form of diabetes. Nat. Genet. *45*, 1380–1385.
7. Gaulton, K.J., Ferreira, T., Lee, Y., Raimondo, A., Mägi, R., Reschen, M.E., Mahajan, A., Locke, A., Rayner, N.W., Robertson, N., et al.; DIAbetes Genetics Replication And Meta-analysis (DIAGRAM) Consortium (2015). Genetic fine mapping and genomic annotation defines causal mechanisms at type 2 diabetes susceptibility loci. Nat. Genet. *47*, 1415–1425.
8. Sagen, J.V., Bjørkhaug, L., Haukanes, B.I., Grevle, L., Molnes, J., Nedrebø, B.G., Søvik, O., Njølstad, P.R., Johansson, S., and Molven, A. (2017). The HNF1A mutant Ala180Val: Clinical challenges in determining causality of a rare HNF1A variant in familial diabetes. Diabetes Res. Clin. Pract. *133*, 142–149.
9. Pearson, E.R., Starkey, B.J., Powell, R.J., Gribble, F.M., Clark, P.M., and Hattersley, A.T. (2003). Genetic cause of hyperglycaemia and response to treatment in diabetes. Lancet *362*, 1275–1281.
10. Shepherd, M.H., Shields, B.M., Hudson, M., Pearson, E.R., Hyde, C., Ellard, S., Hattersley, A.T., Patel, K.A.; and UNITED study (2018). A UK nationwide prospective study of treatment change in MODY: genetic subtype and clinical characteristics predict optimal glycaemic control after discontinuing insulin and metformin. Diabetologia *61*, 2520–2527.
11. Cooper, G.M. (2015). Parlez-vous VUS? Genome Res. *25*, 1423–1426.
12. Richards, S., Aziz, N., Bale, S., Bick, D., Das, S., Gastier-Foster, J., Grody, W.W., Hegde, M., Lyon, E., Spector, E., et al.; ACMG Laboratory Quality Assurance Committee (2015). Standards and guidelines for the interpretation of sequence variants: a joint consensus recommendation of the American College of Medical Genetics and Genomics and the Association for Molecular Pathology. Genet. Med. *17*, 405–424.
13. Harries, L.W., Ellard, S., Stride, A., Morgan, N.G., and Hattersley, A.T. (2006). Isomers of the TCF1 gene encoding hepatocyte nuclear factor-1 alpha show differential expression in the pancreas and define the relationship between mutation position and clinical phenotype in monogenic diabetes. Hum. Mol. Genet. *15*, 2216–2224.
14. Bellanné-Chantelot, C., Carette, C., Riveline, J.P., Valéro, R., Gautier, J.F., Larger, E., Reznik, Y., Ducluzeau, P.H., Sola, A., Hartemann-Heurtier, A., et al. (2008). The type and the position of HNF1A mutation modulate age at diagnosis of diabetes in patients with maturity-onset diabetes of the young (MODY)-3. Diabetes *57*, 503–508.
15. Fuchsberger, C., Flannick, J., Teslovich, T.M., Mahajan, A., Agarwala, V., Gaulton, K.J., Ma, C., Fontanillas, P., Moutsianas, L., McCarthy, D.J., et al. (2016). The genetic architecture of type 2 diabetes. Nature *536*, 41–47.
16. Kumar, P., Henikoff, S., and Ng, P.C. (2009). Predicting the effects of coding non-synonymous variants on protein function using the SIFT algorithm. Nat. Protoc. *4*, 1073–1081.
17. Adzhubei, I., Jordan, D.M., and Sunyaev, S.R. (2013). Predicting functional effect of human missense mutations using PolyPhen-2. Curr. Protoc. Hum. Genet, Chapter 7: Unit7 20.
18. Schwarz, J.M., Rödelsperger, C., Schuelke, M., and Seelow, D. (2010). MutationTaster evaluates disease-causing potential of sequence alterations. Nat. Methods *7*, 575–576.
19. Kircher, M., Witten, D.M., Jain, P., O'Roak, B.J., Cooper, G.M., and Shendure, J. (2014). A general framework for estimating the relative pathogenicity of human genetic variants. Nat. Genet. *46*, 310–315.
20. Bjørkhaug, L., Sagen, J.V., Thorsby, P., Søvik, O., Molven, A., and Njølstad, P.R. (2003). Hepatocyte nuclear factor-1 alpha gene mutations and diabetes in Norway. J. Clin. Endocrinol. Metab. *88*, 920–931.
21. Bjørkhaug, L., Molnes, J., Søvik, O., Njølstad, P.R., and Flatmark, T. (2007). Allosteric activation of human glucokinase by free polyubiquitin chains and its ubiquitin-dependent cotranslational proteasomal degradation. J. Biol. Chem. *282*, 22757–22764.
22. Kaci, A., Keindl, M., Solheim, M.H., Njølstad, P.R., Bjørkhaug, L., and Aukrust, I. (2018). The E3 SUMO ligase PIASγ is a novel interaction partner regulating the activity of diabetes associated hepatocyte nuclear factor-1α. Sci. Rep. *8*, 12780.
23. Bjørkhaug, L., Bratland, A., Njølstad, P.R., and Molven, A. (2005). Functional dissection of the HNF-1alpha transcription factor: a study on nuclear localization and transcriptional activation. DNA Cell Biol. *24*, 661–669.
24. Bjørkhaug, L., Ye, H., Horikawa, Y., Søvik, O., Molven, A., and Njølstad, P.R. (2000). MODY associated with two novel hepatocyte nuclear factor-1alpha loss-of-function mutations (P112L and Q466X). Biochem. Biophys. Res. Commun. *279*, 792–798.
25. Charrad, M., Ghazzali, N., Boiteau, V., and Niknafs, A. (2014). NbClust: An R Package for Determining the Relevant Number of Clusters in a Data Set. Statistical Software. *61*, 1–36.
26. Johansson, B.B., Irgens, H.U., Molnes, J., Sztromwasser, P., Aukrust, I., Juliusson, P.B., Søvik, O., Levy, S., Skrivarhaug, T., Joner, G., et al. (2017). Targeted next-generation sequencing reveals MODY in up to 6.5% of antibody-negative diabetes cases listed in the Norwegian Childhood Diabetes Registry. Diabetologia *60*, 625–635.
27. Plon, S.E., Eccles, D.M., Easton, D., Foulkes, W.D., Genuardi, M., Greenblatt, M.S., Hogervorst, F.B., Hoogerbrugge, N., Spurdle, A.B., Tavtigian, S.V.; and IARC Unclassified Genetic Variants Working Group (2008). Sequence variant classification and reporting: recommendations for improving the interpretation of cancer susceptibility genetic test results. Hum. Mutat. *29*, 1282–1291.
28. McDonald, T.J., and Ellard, S. (2013). Maturity onset diabetes of the young: identification and diagnosis. Ann. Clin. Biochem. *50*, 403–415.
29. Montgomery, S.B., Lappalainen, T., Gutierrez-Arcelus, M., and Dermitzakis, E.T. (2011). Rare and common regulatory variation in population-scale sequenced human genomes. PLoS Genet. *7*, e1002144.
30. Castel, S.E., Cervera, A., Mohammadi, P., Aguet, F., Reverter, F., Wolman, A., Guigo, R., Iossifov, I., Vasileva, A., and Lappalainen, T. (2018). Modified penetrance of coding variants by cis-regulatory variation contributes to disease risk. Nat. Genet. *50*, 1327–1334.
31. Manning, K.S., and Cooper, T.A. (2017). The roles of RNA processing in translating genotype to phenotype. Nat. Rev. Mol. Cell Biol. *18*, 102–114.

32. Nickels, S., Truong, T., Hein, R., Stevens, K., Buck, K., Behrens, S., Eilber, U., Schmidt, M., Häberle, L., Vrieling, A., et al.; Genica Network; kConFab; and AOCS Management Group (2013). Evidence of gene-environment interactions between common breast cancer susceptibility loci and established environmental risk factors. PLoS Genet. *9*, e1003284.
33. Locke, J.M., Saint-Martin, C., Laver, T.W., Patel, K.A., Wood, A.R., Sharp, S.A., Ellard, S., Bellanné-Chantelot, C., Hattersley, A.T., Harries, L.W., and Weedon, M.N. (2018). The common HNF1A variant I27L is a modifier of age at diabetes diagnosis in individuals with HNF1A-MODY. Diabetes *67*, 1903–1907.
34. Flannick, J., Johansson, S., and Njølstad, P.R. (2016). Common and rare forms of diabetes mellitus: towards a continuum of diabetes subtypes. Nat. Rev. Endocrinol. *12*, 394–406.
35. Starita, L.M., Ahituv, N., Dunham, M.J., Kitzman, J.O., Roth, F.P., Seelig, G., Shendure, J., and Fowler, D.M. (2017). Variant Interpretation: Functional Assays to the Rescue. Am. J. Hum. Genet. *101*, 315–325.

The American Journal of Human Genetics, Volume *107*

Supplemental Data

Unsupervised Clustering of Missense Variants in *HNF1A* Using Multidimensional Functional Data Aids Clinical Interpretation

Sara Althari, Laeya A. Najmi, Amanda J. Bennett, Ingvild Aukrust, Jana K. Rundle, Kevin Colclough, Janne Molnes, Alba Kaci, Sameena Nawaz, Timme van der Lugt, Neelam Hassanali, Anubha Mahajan, Anders Molven, Sian Ellard, Mark I. McCarthy, Lise Bjørkhaug, Pål Rasmus Njølstad, and Anna L. Gloyn

SUPPLEMENTAL DATA

Contents

Figure S1. Ethnic composition of a multi-consortia led exome sequencing study

Figure S2. Division of exome-identified non-synonymous missense alleles

Figure S3. Analytical pipeline for unsupervised stratification of HNF1A missense alleles along the in vivo glycaemic spectrum using multi-dimensional in vitro functional data

Figure S4. Assessment of the optimal number of clusters

Figure S5. Functional studies of protein variants (Bergen)

Figure S6. Functional studies of protein variants (Oxford)

Figure S7. Clustering alignment of *HNF1A* missense variants shared between both centres at Oxford and Bergen

Table S1. Exome-detected *HNF1A* missense alleles

Table S2. MODY reference alleles

Table S3. Scores for tissue specific isoform expression and functional domain

Table S4. Features of *HNF1A* allele carriers documented in UK Registry

Table S5. Features of *HNF1A* allele carriers documented in Norwegian Registry

Table S6. Clinical features discordant *HNF1A* alleles in UK Registry

Table S7. Clinical features discordant *HNF1A* alleles in Norwegian Registry

A

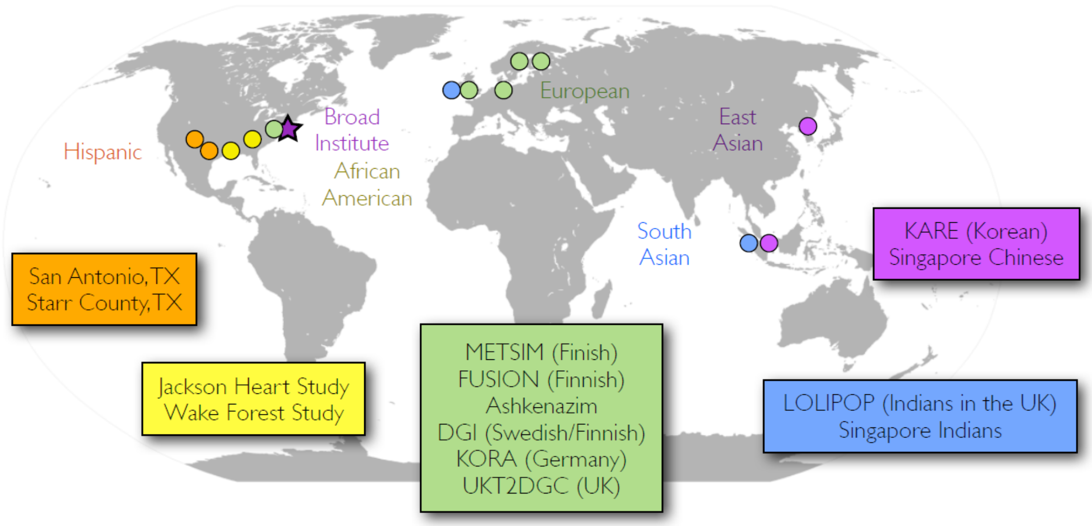

B

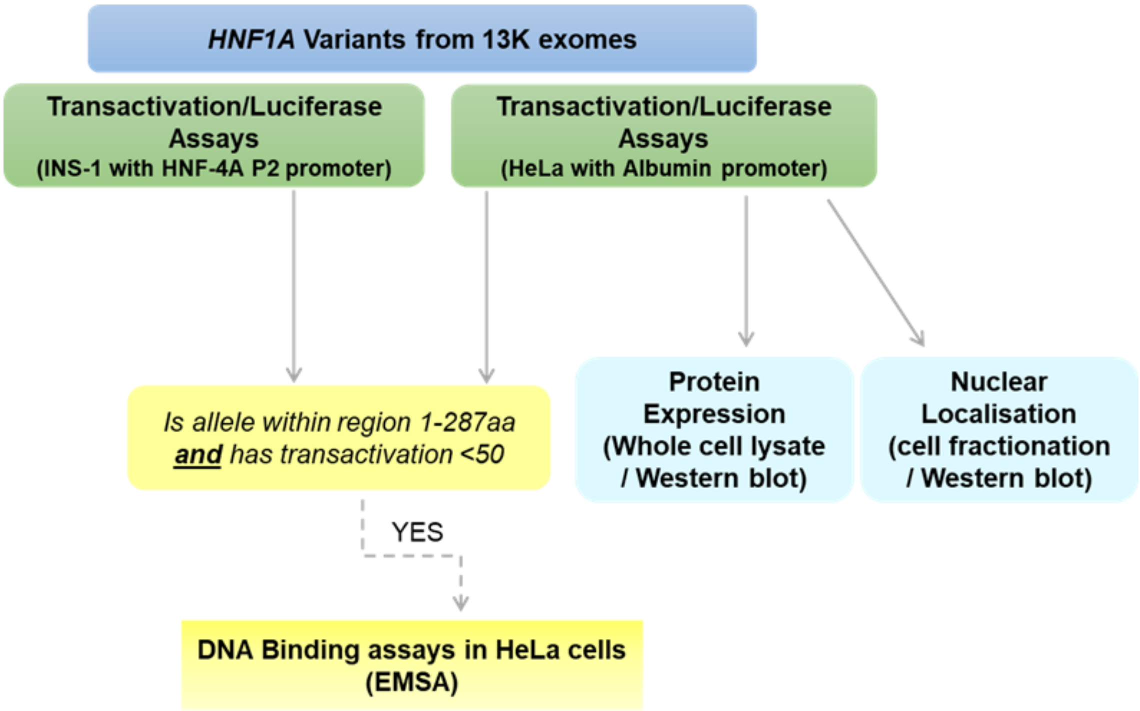

Figure S1. Ethnic Composition of a Multi-Consortia Led Exome Sequencing Study

(A). Molecular characterisation pipeline adopted by research groups at Oxford and Bergen to annotate the function of exome-detected *HNF1A* missense variants (B). Exome sequence data was generated from ~13K individuals (6,504 type 2 diabetes cases and 6,436 controls) from five ancestry groups with 82x mean coverage across the protein coding sequence of 18,281 genes, identifying 3.04 million variants (1.19 million protein-altering).[15]

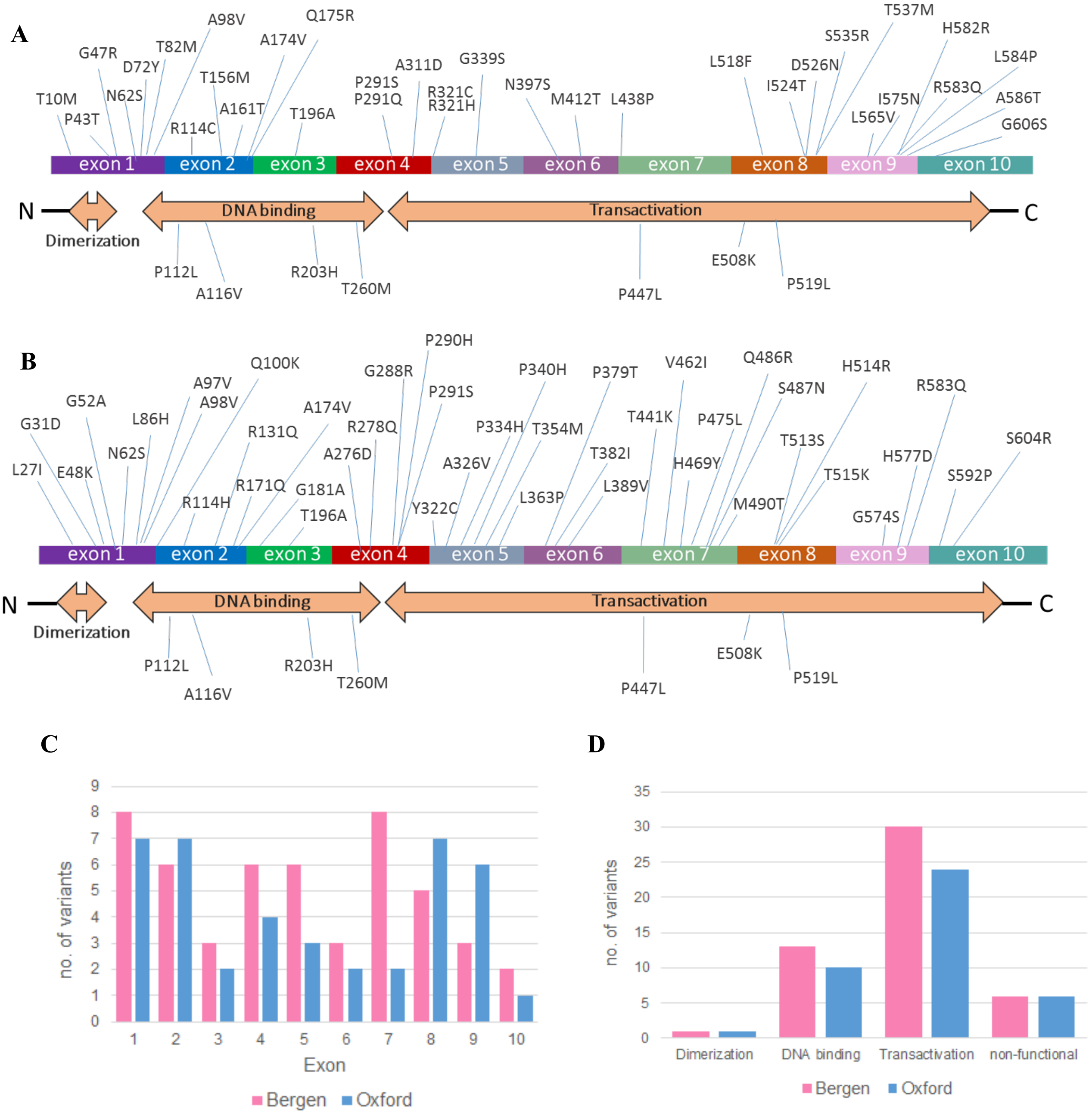

Figure S2. Division of Exome-Identified Non-Synonymous Missense Alleles in *HNF1A* Between Oxford and Bergen for Large-Scale Collaborative Functional Follow-up(A) Oxford variant set **(B)** Bergen variant set **(C)** Distribution of exome-detected *HNF1A* variants by centre based on exon position **(D)** Distribution of exome-detected *HNF1A* alleles by centre based on functional domain.

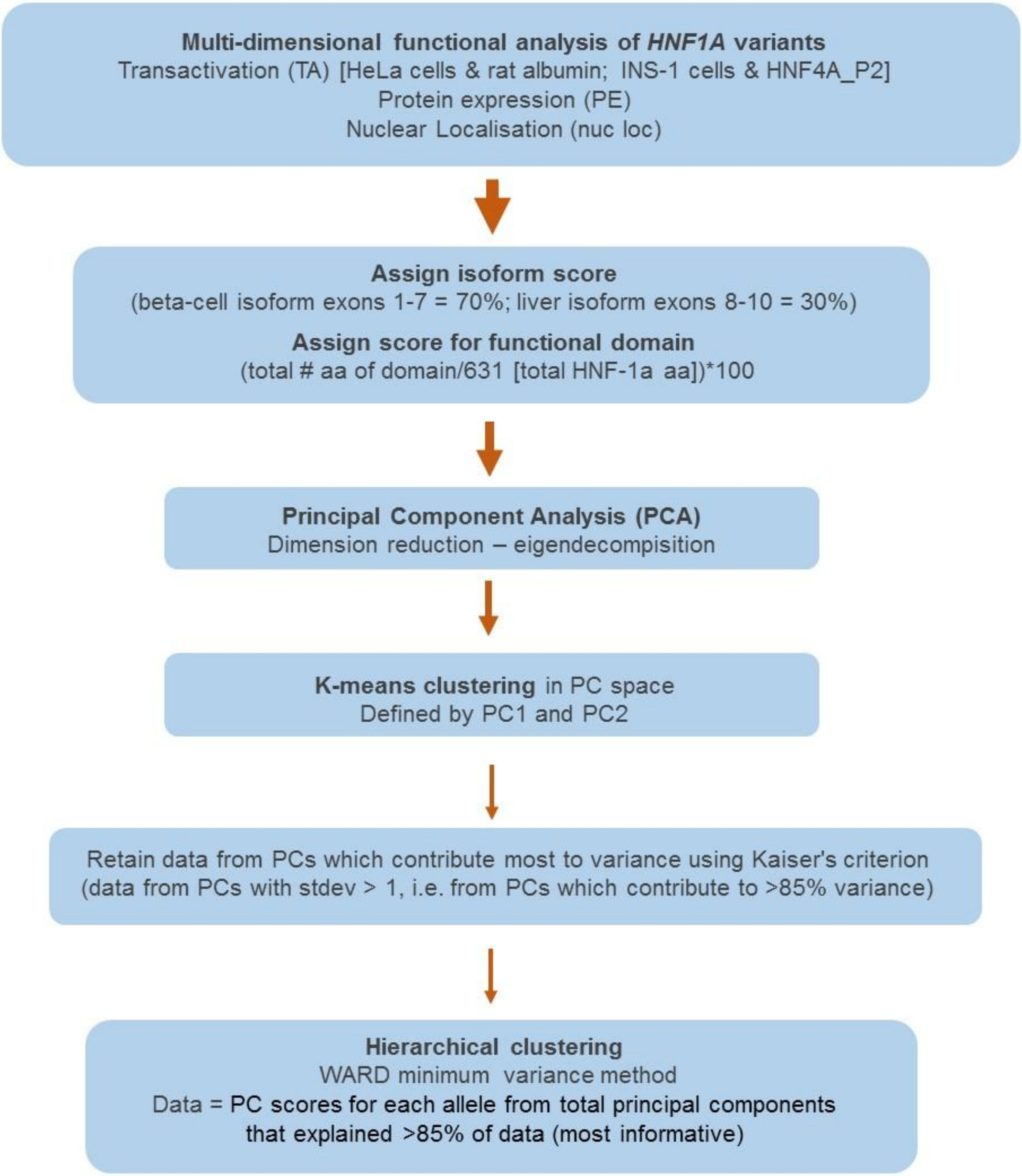

Figure S3. Analytical pipeline for unsupervised stratification of HNF1A missense variants along the in vivo glycaemic spectrum using multi-dimensional in vitro functional data. The pipeline we developed to perform unbiased classification of functionally characterised missense alleles in HNF1A is as follows: 1) dataset preparation (omitting missing values and unevenly represented functional parameters, and selecting the optimal input data format), 2) assignment of weights for tissue-specific isoform expression (liver v pancreatic beta-cell dominant isoforms) and functional domain (dimerization, undefined, DNA binding, transactivation), 3) Dimension reduction by principal component analysis, 4) retention of values from principal components which contribute the most to data variance according to Kaiser's criterion (>85% explained variance by eigenvalues i.e. all PCs with standard deviation > 1) to avoid fitting noise, 5) hierarchical clustering analysis of data points from PCs retained in step 4 using WARD minimum variance method. Abbreviations: aa= amino acids; stdev = standard deviation; PCs = principal components.

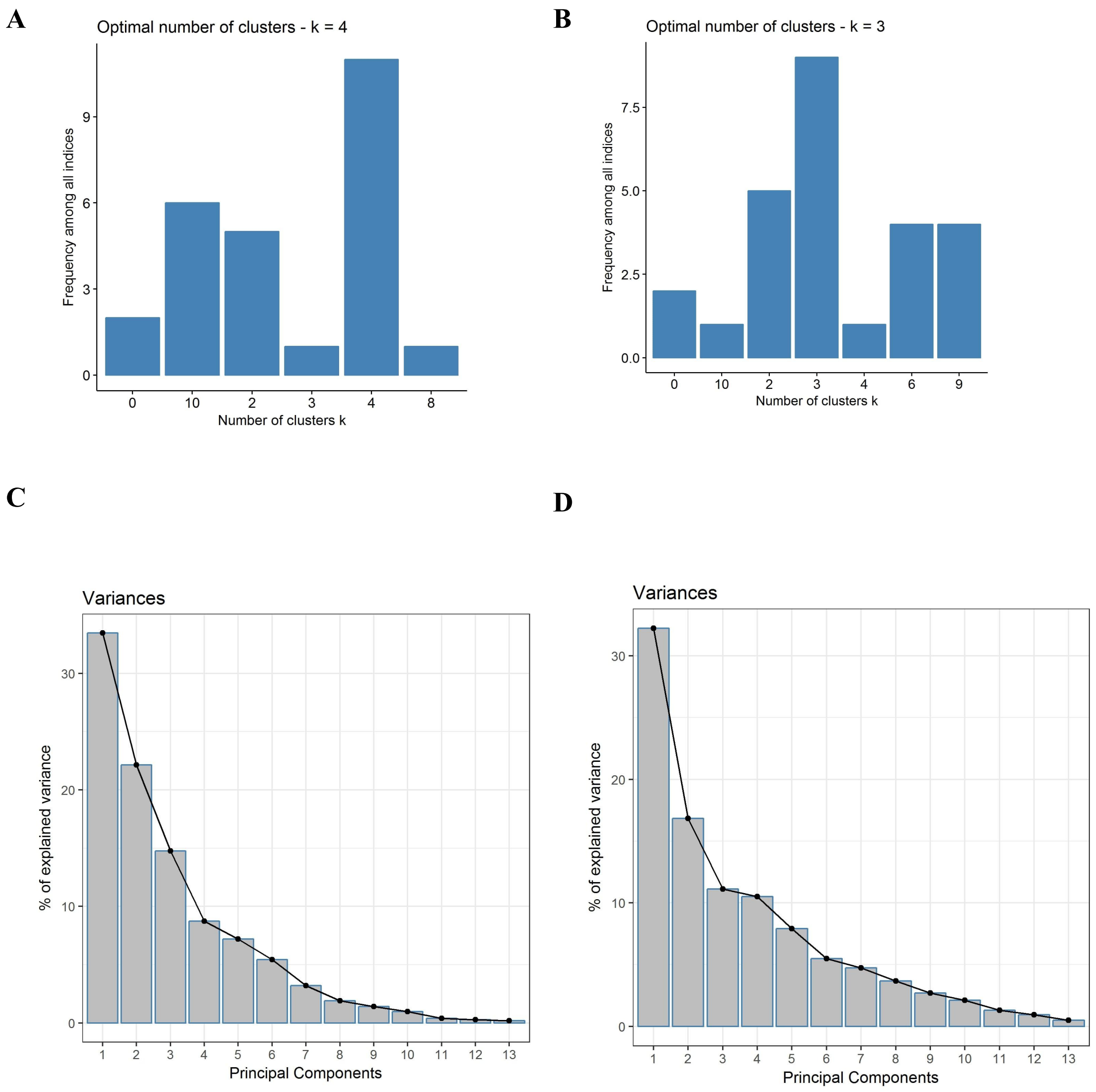

Figure S4. Assessment of the Optimal Number of Clusters to Use for Partitioning the Multi-Dimensional Datasets. Dataset generated at (A) Oxford and (B) Bergen. Best cluster scheme obtained using *NbClust* R package which provides majority rule across 30 tested indices using euclidean distance. **Scree plots showing percent explained variance from PCA of Oxford (C) and Bergen (D) datasets.**

Summary of Figure S5 and S6 - Functional studies of Protein Variants

The variants studied at both Centers included five exome variants (p.Asn62Ser, p.Ala174Val, p.Pro291Ser, p.Thr196Ala, p.Arg583Gln), two type 2 risk variants (p.Ala98Val, p.Glu508Lys), and six MODY variants (super controls; p.Ala116Val, p.Arg203His, p.Pro112Leu, p.Pro447Leu, p.Thr260Met, p.Pro519Leu), with single variants missing in some individual assays.

Correlation of functional effects between centers - For the transactivation assay, testing two promoters in different cell lines (HeLa/INS), the control variants correlated well between centers by range of effect; the MODY control variants presented the most severe effect, and the type 2 diabetes risk variants medium effects (deviation by the p.Ala116Val MODY control variant in one promoter assay). For the exome variants, the functional effect also correlated between centers, i.e. less severe effect than the rare type 2 risk variant (p.Glu508Lys) (deviation p.Pro291Ser between Centers). Overall, values of all tested variants were somewhat lower at one center (Bergen).

For the subsequent DNA binding assay, only one mutual exome variant was tested at both centers (p.Ala174Val), revealing correlating effects between the two centers. The same applied to the two MODY variants, although lower values (more severe effect) were detected at one center (Oxford).

For the protein expression assay, the MODY and type 2 risk variants correlated well between the centers (deviation for p.Glu508Lys exhibiting stronger reducing effect by one center (Bergen <0.6 fold versus Oxford WT-like). Two (p.Asn62Ser, p.Arg583Gln) of the five exome variants also deviated between the two centers (Oxford ~0.6 fold versus Bergen WT-like).

The nuclear localization assay correlated less between the centers. Two (p.Pro447Leu, p.Ala116Val) of six MODY variants, the two type 2 risk variants (p.Ala98Val, p.Glu508Lys), and the two exome variants (p.Ala174Val, p.Arg583Gln) did not correlate by range of effect (Bergen by stronger reducing effect compared to Oxford). The non-patient related control variant included as the best indicator of strongly reduced nuclear localization (DelB) did, however, correlate well between centers.

Of the four functional assays, it was not unexpected that functional effect between centers correlated best for the transactivation and DNA binding assays, whereas the protein expression and nuclear localization assays had more variable effect sizes when compared. This latter is most likely due several experimental steps prior to final quantifications, which is based on immunoblotting and antibody specificity.

A

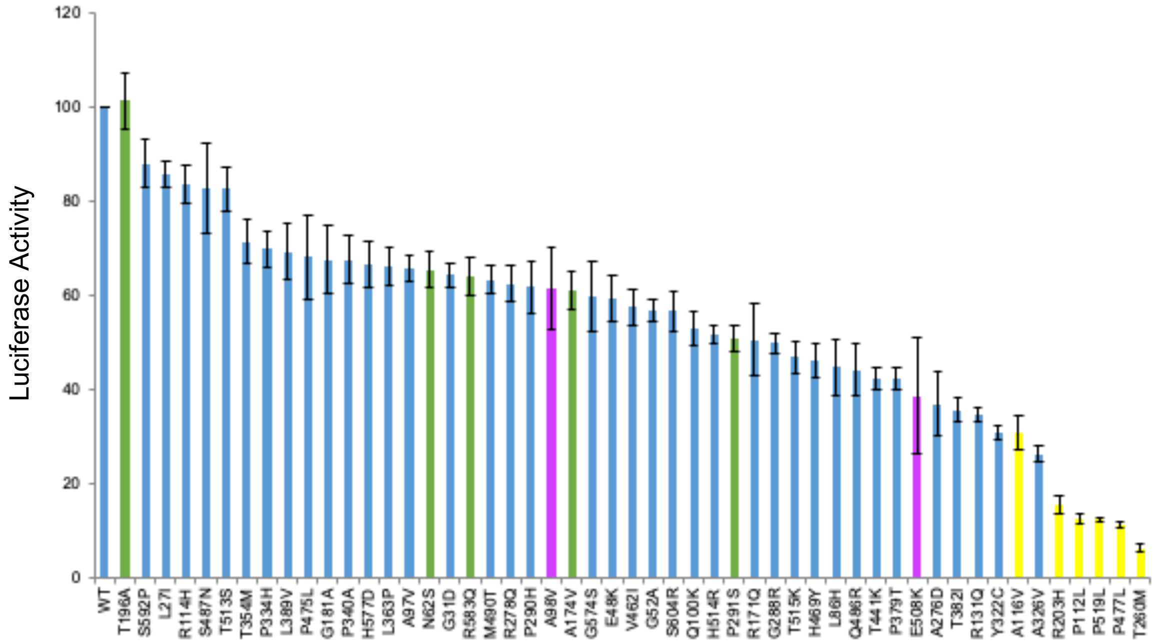
Luciferase Activity
0
20
40
60
80
100
120
WT
T196A
S592P
L27I
R114H
S487N
T513S
T354M
P334H
L389V
P475L
G181A
P340A
H577D
L363P
A97V
N62S
G31D
R583Q
M490T
R278Q
P290H
A98V
A174V
G574S
E48K
V462I
G52A
S604R
Q100K
H514R
P291S
R171Q
G288R
T515K
H469Y
L86H
Q486R
T441K
P379T
E508K
A276D
T382I
R131Q
Y322C
A116V
A326V
R203H
P112L
P519L
P477L
T260M

B

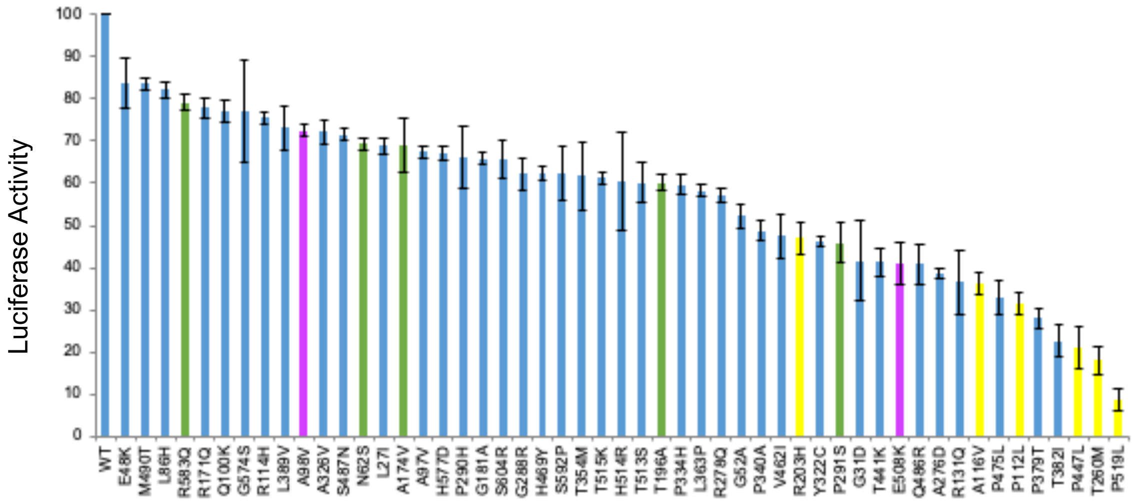
Luciferase Activity
0
10
20
30
40
50
60
70
80
90
100
WT
E48K
M490T
L86H
R583Q
R171Q
Q100K
G574S
R114H
L389V
A98V
A326V
S487N
N62S
L27I
A174V
A97V
H577D
P290H
G181A
S604R
G288R
H469Y
S592P
T354M
T515K
H514R
T513S
T196A
P334H
L363P
R278Q
G52A
P340A
V462I
R203H
Y322C
P291S
G31D
T441K
E508K
Q486R
A276D
R131Q
A116V
P475L
P112L
P379T
T382I
P447L
T260M
P519L

C

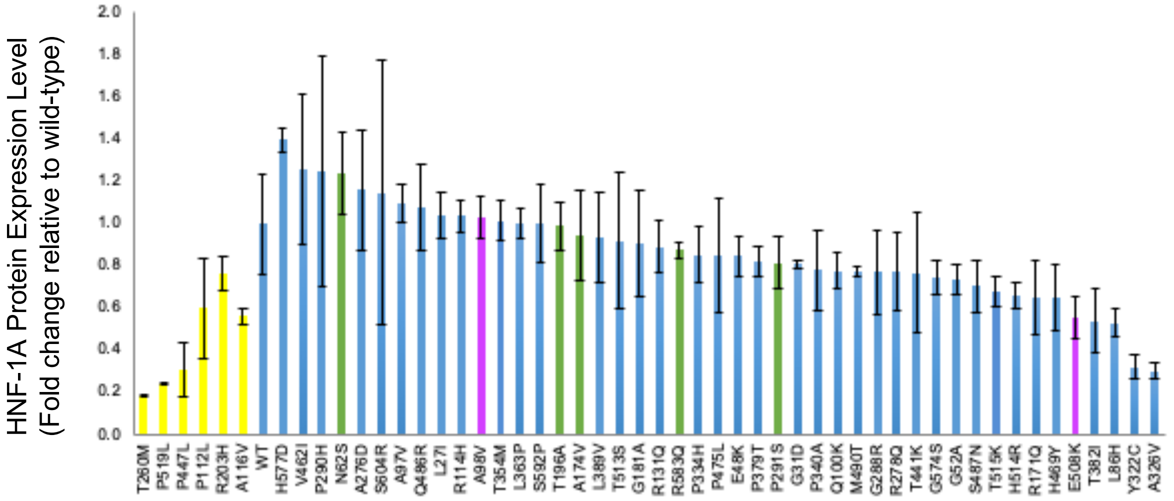
HNF-1A Protein Expression Level
(Fold change relative to wild-type)
2.0
1.8
1.6
1.4
1.2
1.0
0.8
0.6
0.4
0.2
0.0
T260M
P519L
P447L
P112L
R203H
A116V
WT
H577D
V462I
P290H
N62S
A276D
S604R
A97V
Q486R
L27I
R114H
A98V
T354M
L363P
S592P
T196A
A174V
L389V
T513S
G181A
R131Q
R583Q
P334H
P475L
E48K
P379T
P291S
G31D
P340A
Q100K
M490T
G288R
R278Q
T441K
G574S
G52A
S487N
T515K
H514R
R171Q
H469Y
E508K
T382I
L86H
Y322C
A326V

D

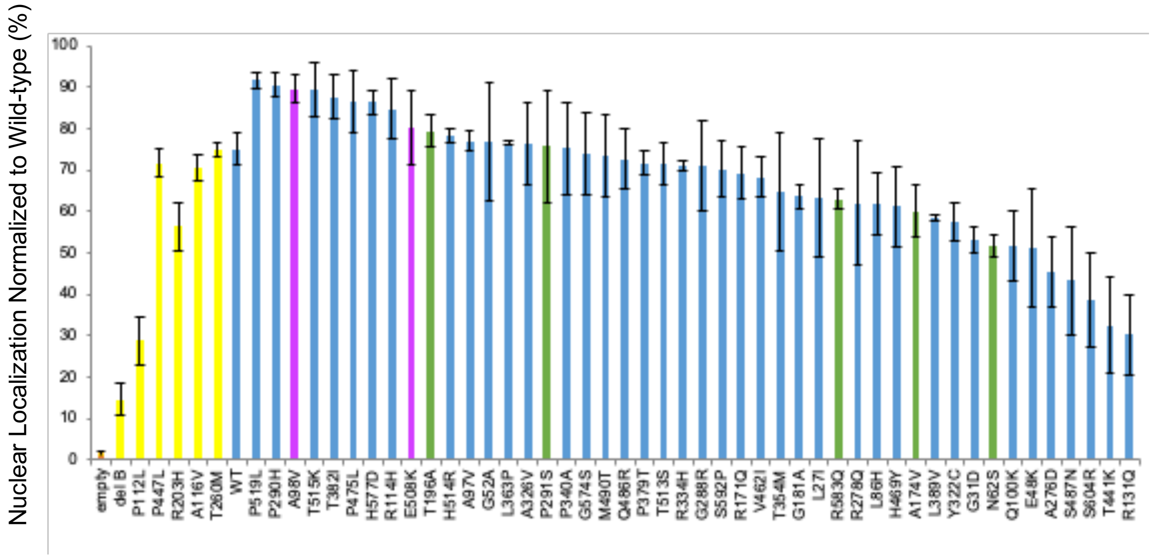
Nuclear Localization Normalized to Wild-type (%)
100
90
80
70
60
50
40
30
20
10
0
empty
del B
P112L
P447L
R203H
A116V
T260M
WT
P519L
P290H
A98V
T515K
T382I
P475L
H577D
R114H
E508K
T196A
H514R
A97V
G52A
L363P
A326V
P291S
P340A
G574S
M490T
Q486R
P379T
T513S
R334H
G288R
S592P
R171Q
V462I
T354M
G181A
L27I
R583Q
R278Q
L86H
H469Y
A174V
L389V
Y322C
G31D
N62S
Q100K
E48K
A276D
S487N
S604R
T441K
R131Q

E

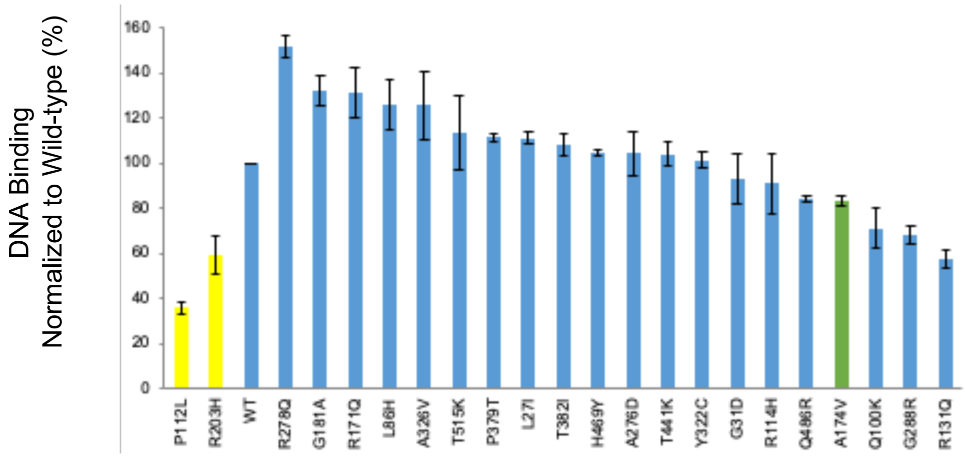

Figure S5. Functional Studies of Protein Variants (Bergen)

A and B) Transcriptional activity of HNF-1A protein variants measured by luciferase reporter assay. Transcriptional activity was performed in two individual cell lines using two different promoter-linked luciferase reporter constructs. A) HeLa cell line using rat albumin promoter and B) INS-1 cell line using HNF4AP2 promoter. Cells were transiently transfected with wild-type or variant *HNF1A* variant plasmids together with reporter plasmids pGL3-RA (Firefly Luciferase under rat albumin promoter) or pGL3-HNF4AP2 (Firefly Luciferase under HNF4AP2 promoter), and pRL-SV40 (Renilla Luciferase as internal control). Cells were harvested/lysed 24 h after transfection and Firefly activity recorded after normalizing for Renilla activity. HNF-1A variant measurements are given in percentage of wild-type activity (100%). Each bar represents the mean of nine readings ± SD; three parallel readings were conducted on each of 3 experimental days (n = 3). Different colors are used for different group of variants; shared control variants are shown in green, type 2 diabetes associated variants in pink and MODY control variants in yellow.

C) Variant effect on HNF-1A protein expression level. The level of expression of HNF-1A variant proteins was assessed relative to wild-type protein level in cells. For this purpose, 20 µl HeLa cell lysates prepared for transactivation assay (panel A) was analyzed by SDS-PAGE and immunoblotting using an HNF-1A specific antibody. A house-keeping protein (actin) was used as internal control for normalization and bands were quantitated using densitometric analysis by Quantity One 1-D software. Each bar represents the level of HNF-1A protein expression normalized to wild-type HNF-1A expression level (100%). Different colors are used for different group of variants; shared control variants are shown in green, type 2 diabetes associated variants in pink and MODY control variants in yellow.

D) Nuclear localization of HNF-1A variant proteins. The effect of individual *HNF1A* variants on HNF-1A protein localization (nuclear versus cytosolic) was assessed in cells. For this purpose, HeLa cells were cultured and transiently transfected with wild-type or *HNF1A* variant plasmids and sequential cell fractionation was performed 24h post-transfection by isolating the nuclear and cytosol fractions from each transfected sample. 20 µg total protein from each isolated compartment was analyzed for HNF-1A expression by SDS-PAGE and immunoblotting using an HNF-1A specific antibody. HNF-1A variant p.delB was included as a positive control for impaired nuclear localization (cytosolic retention). HNF-1A variant nuclear localization measurements are given in percentage of wild-type localization. Each bar represents the mean of nuclear localization of HNF-1A protein of two biological replicates in two experimental days (n = 2). Different colors are used for different group of variants; shared control variants are shown in green, type 2 diabetes associated variants in pink and MODY control variants in yellow.

E) DNA binding of HNF-1A protein variants to the rat albumin promoter as studied by EMSA. DNA binding ability test was conducted for HNF-1A variants that are either located in DNA binding domain (1-287 aa) or those that demonstrated transactivation activity < 50%. Electrophoretic mobility shift assay (EMSA) was performed to investigate the DNA binding ability of equal amounts of *in vitro*-synthesized HNF-1A proteins to a ^{32}P-radiolabeled rat albumin oligonucleotide containing an HNF-1A binding site. A coupled *in vitro* transcription and translation system (TNT) was used for expression of HNF-1A proteins. DNA-protein bound complexes were analyzed by DNA retardation gel (6%) electrophoresis followed by autoradiography. Level of DNA binding was obtained by quantification of the intensity of HNF-1A protein-oligonucleotide complexes. Measurements are given in percentage of wild-type binding activity (100%). Two *HNF1A*-MODY3 variants were used as control for low binding (yellow) and shared control variants are presented in green color.

A

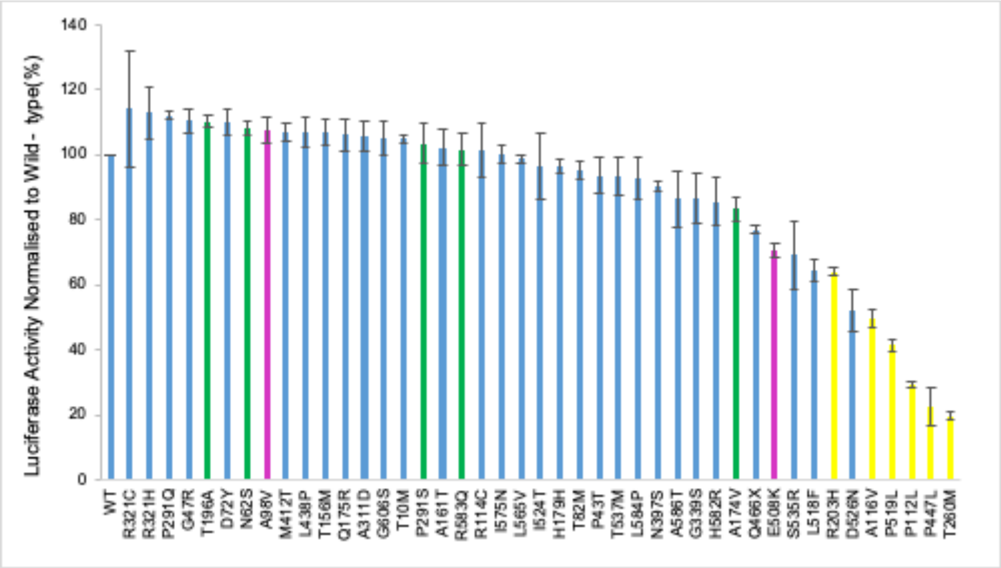
Luciferase Activity Normalised to Wild - type(%)
140
120
100
80
60
40
20
0
WT
R321C
R321H
P291Q
G47R
T196A
D72Y
N62S
A98V
M412T
L438P
T156M
Q175R
A311D
G606S
T10M
P291S
A161T
R583Q
R114C
I575N
L565V
I524T
H179H
T82M
P43T
T537M
L584P
N397S
A586T
G339S
H582R
A174V
Q466X
E508K
S535R
L518F
R203H
D526N
A116V
P519L
P112L
P447L
T260M

B

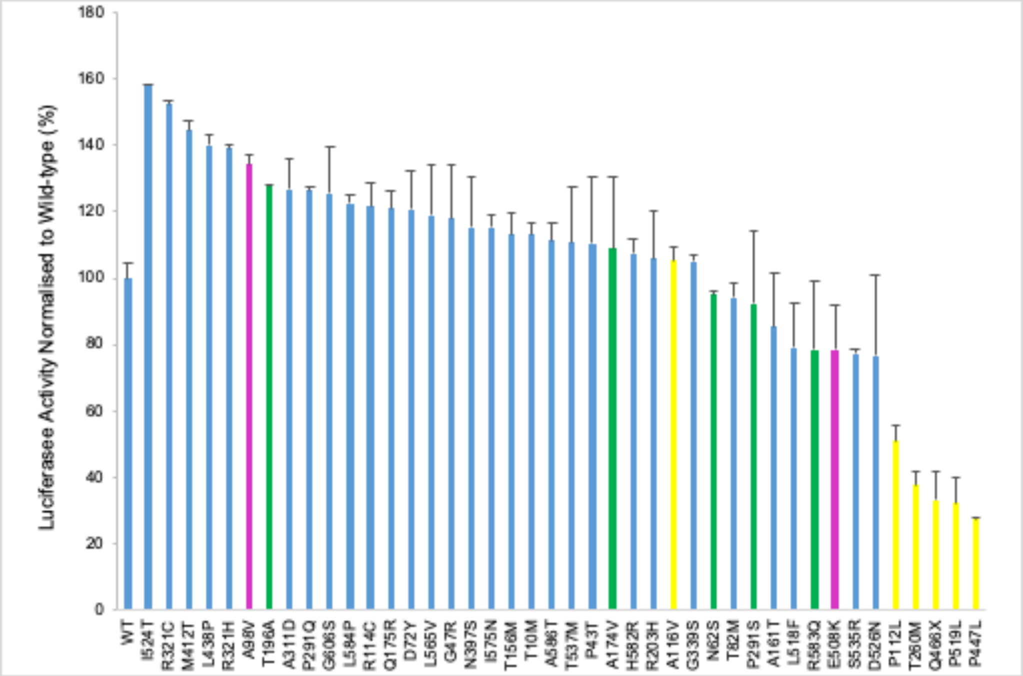
Luciferasee Activity Normalised to Wild-type (%)
180
160
140
120
100
80
60
40
20
0
WT
I524T
R321C
M412T
L438P
R321H
A98V
T196A
A311D
P291Q
G606S
L584P
R114C
Q175R
D72Y
L565V
G47R
N397S
I575N
T156M
T10M
A586T
T537M
P43T
A174V
H582R
R203H
A116V
G339S
N62S
T82M
P291S
A161T
L518F
R583Q
E508K
S535R
D526N
P112L
T260M
Q466X
P519L
P447L

C

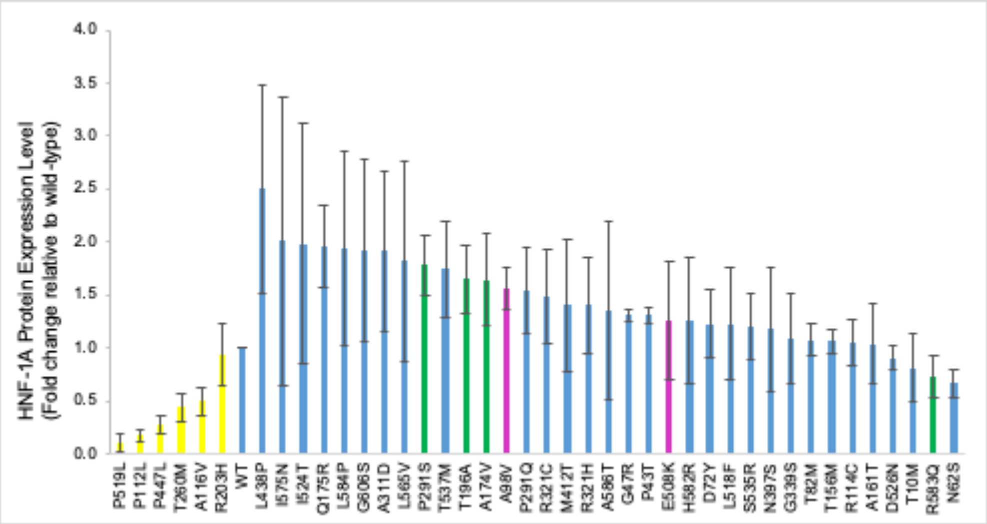
HNF-1A Protein Expression Level
(Fold change relative to wild-type)
4.0
3.5
3.0
2.5
2.0
1.5
1.0
0.5
0.0
P519L
P112L
P447L
T260M
A116V
R203H
WT
L438P
I575N
I524T
Q175R
L584P
G606S
A311D
L565V
P291S
T537M
T196A
A174V
A98V
P291Q
R321C
M412T
R321H
A586T
G47R
P43T
E508K
H582R
D72Y
L518F
S535R
N397S
G339S
T82M
T156M
R114C
A161T
D526N
T10M
R583Q
N62S

D

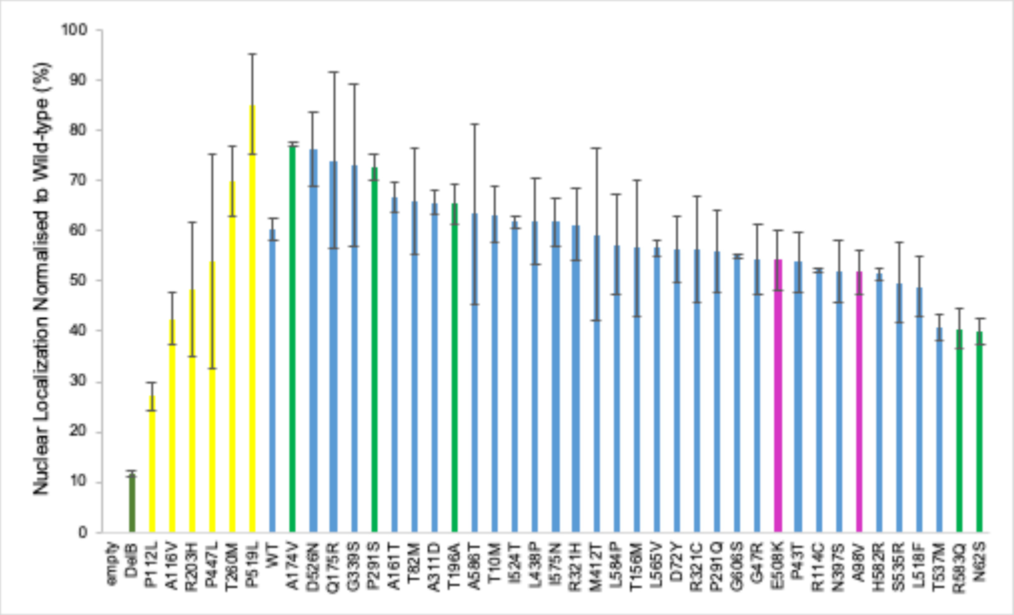
Nuclear Localization Normalised to Wild-type (%)
100
90
80
70
60
50
40
30
20
10
0
empty
DelB
P112L
A116V
R203H
P447L
T260M
P519L
WT
A174V
D526N
Q175R
G339S
P291S
A161T
T82M
A311D
T196A
A586T
T10M
I524T
L438P
I575N
R321H
M412T
L584P
T156M
L565V
D72Y
R321C
P291Q
G606S
G47R
E508K
P43T
R114C
N397S
A98V
H582R
S535R
L518F
T537M
R583Q
N62S

E

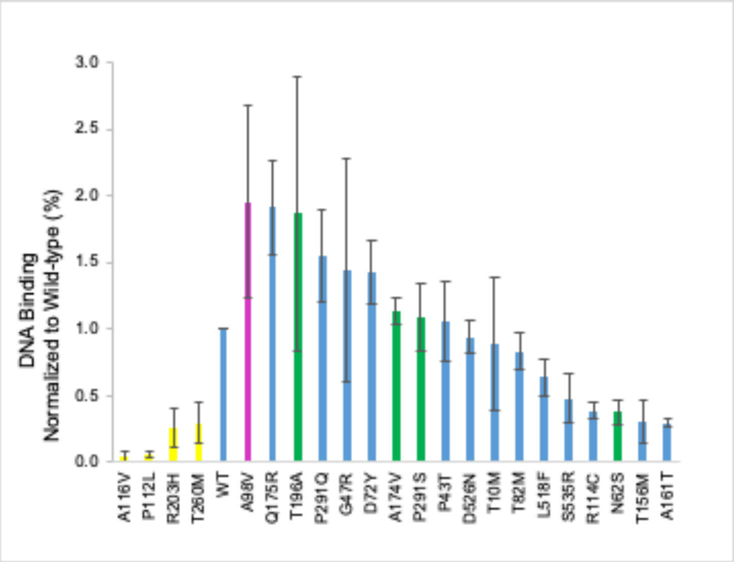

Figure S6. Functional Studies of Protein Variants (Oxford)

A and B) Transcriptional activity of HNF-1A protein variants measured by luciferase reporter assay. Transcriptional activity was performed in two individual cell lines using two different promoter-linked luciferase reporter constructs. A) HeLa cell line using rat albumin promoter and B) INS-1 cell line using *HNF4A* P2 promoter. Cells were transiently transfected with wild-type or variant *HNF1A* variant plasmids together with reporter plasmids pGL3-RA (Firefly Luciferase under rat albumin promoter) or pGL3-*HNF4A* P2 (Firefly Luciferase under *HNF4A* P2 promoter), and pRL-SV40 (Renilla Luciferase as internal control). Cells were harvested/lysed 24 h after transfection and Firefly activity recorded after normalizing for Renilla activity. HNF-1A variant measurements are given in percentage of wild-type activity (100%). Each bar represents the mean of nine readings ± SD; three parallel readings were conducted on each of three experimental days (n = 3). Different colors are used for different group of variants; shared control variants are shown in green, type 2 diabetes associated variants in pink and MODY control variants in yellow.

C) Variant effect on HNF-1A protein expression level. The level of expression of HNF-1A variant proteins was assessed relative to wild-type protein level in cells. For this purpose, HeLa cells were cultured and transiently transfected with wild-type or *HNF1A* variant plasmids. Cells were harvested at 24 hrs and aliquoted prior to lysing for the protein expression levels and EMSA or nuclear localization. 10 µg of HeLa cell lysates prepared for transactivation assay (Figure A) was analyzed by SDS-PAGE and immunobloting using an HNF-1A specific antibody. A house-keeping protein (B-tubulin) was used as internal control for normalization of levels of tubulin versus HNF-1A specific bands., using densitometric analysis by Image Lab software (Bio-Rad). Each bar represents the level of HNF-1A protein expression normalized to wild-type HNF-1A expression level (100%). Different colors are used for different groups of variants; shared control variants are shown in green, type 2 diabetes associated variants in pink and MODY control variants in yellow.

D) Nuclear localization of HNF-1A variant proteins. The effect of individual *HNF1A* variants on HNF-1A protein localization (nuclear versus cytosolic) was assessed in cells. For this purpose, an aliquot of the HeLa cells that had been transfected and harvested at 24hr were lysed to isolate the nuclear and cytosol fraction. 20 µg total protein from each isolated compartment was analyzed for HNF-1A expression by SDS-PAGE and immunobloting using an HNF-1A specific antibody. HNF-1A variant p.delB was included as a positive control for impaired nuclear localization (cytosolic retention). HNF-1A variant nuclear localization measurements are given in percentage of wild-type localization. Each bar represents the mean of nuclear

localization of HNF-1A protein of two biological replicates in two experimental days (*n* = 2). Different colors are used for different groups of variants; shared control variants are shown in green, type 2 diabetes associated variants in pink and MODY control variants in yellow.

E) DNA binding of HNF-1A protein variants to the rat albumin promoter as studied by EMSA. DNA binding ability test was conducted for *HNF1A* variants that are either located in DNA binding domain (1-287 aa) or those that demonstrated transactivation activity < 50%. Electrophoretic mobility shift assay (EMSA) was performed using an aliquot of the lysed HeLa cells used for the protein expression levels. The HNF-1a wild-type or variant protein was bound to a fluorescently labelled probe and then separated using a TBE polyacrylamide gel. An HNF-1a antibody was used to generate a super-shift with an aliquot of the wild-type protein, prior to the probe binding. Measurements are given as percentage of wild-type binding activity (100%). Different colors are used for different groups of variants; shared control variants are shown in green, type 2 diabetes associated variants in pink and MODY control variants in yellow.

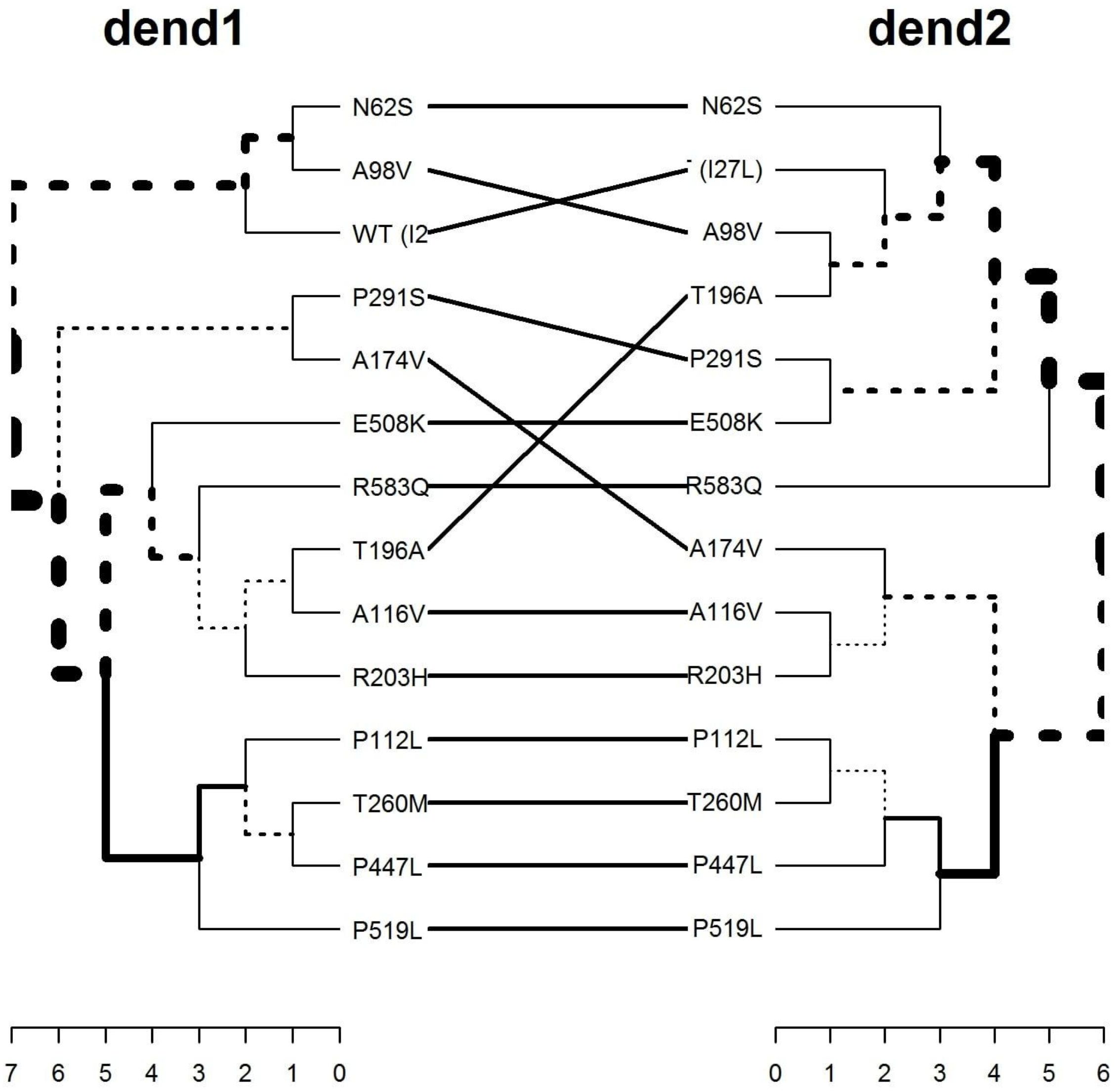

Figure S7. Clustering Alignment of *HNF1A* Missense Variants Shared Between Both Centres at Oxford (dend1and Bergen (dend2). Entanglement (quality of alignment score score from 1 to 0 where lower values = good alignment quality) = 0.055. Dashed lines highlight nodes which contain a combination of alleles not present in the other tree (thickness corresponds to height). The connecting lines highlight subgroups and/or cluster members that are present in both dendrograms. Rotational properties maintained from full variant set dendrogams shown in Figure 3.

Table S1. Exome-Detected *HNF1A* Missense Alleles Selected for Functional Follow-Up (*n* = 73)

(Excel file)

HNF1A reference sequence = NM_000545.6. Colour coding of first three columns (Variant and CDS position NM_0005454): blue = Oxford variant set; red = Bergen variant set; gray = variants shared and characterised by both research groups at Oxford and Bergen. Green highlights variants identified with a prevalence greater than 0.

Table S2. MODY Reference Alleles (*n* = 6)

(Excel file)

Table S3. Scores for Tissue Specific Isoform Expression and Functional Domain

Isoform	Functional Domain
Pancreatic beta-cell isoforms<br>*HNF1A(A)*: exons 1-6<br>UniProtKB Protein isoform identifier P208231-3<br><br>*HNF1A(B)*: exons 1-7<br>UniProtKB Protein isoform identifier P208231-2<br><br>***Hepatic isoform (full length gene)***<br>*HNF1A(C):* exons 1-10<br>NCBI Reference Sequence: NM_000545.8<br>UniProtKB Protein isoform identifier P208231-1	***HNF-1A functional domains***<br>Dimerization = 32 amino acids<br>Undefined = 66 amino acids<br>DNA binding = 187 amino acids<br>Transactivation = 343 amino acids
Weight (%)<br><br>Variants in *HNF1A(A)* and *HNF1A(B)* = [(no. of exons in predominant beta-cell isoforms) ÷ 10 (no. of exons in full length *HNF1A*)] × 100 = 70%<br><br>Variants exclusively present in *HNF1A(C)* = [(no. of additional exons present exclusively in predominant liver isoform) ÷ 10 (no. of exons in full length *HNF1A*)] × 100 = 30%	***Weight (%)***<br><br>[no. of amino acids of domain in which allele is expressed ÷ amino acid length of HNF-1a] × 100<br><br>Dimerization = 4.9%<br>Undefined = 10.45%<br>DNA binding = 29.6%<br>Transactivation = 54.3%

Table S4. Features of *HNF1A* Allele Carriers Documented in UK Registry

Features	Measure
Age at diagnosis	age (years)
Initial Treatment	Diet/OHA/Insulin
Current Treatment	Diet/OHA/Insulin
Sulphonylurea sensitivity	Yes/No
BMI	BMI (kg/m^2)
HbA1c	%
GAD	U/mL
ZnT8	U/mL
IA2	U/mL
C-Peptide	pmol/L
No of generations DM	number of generations with diabetes
Mother DM	Yes/No
Father DM	Yes/No
No of Children DM	number of children with diabetes
Ethnic Origin	ethnicity
Referred to clinic for MODY testing	Yes/No

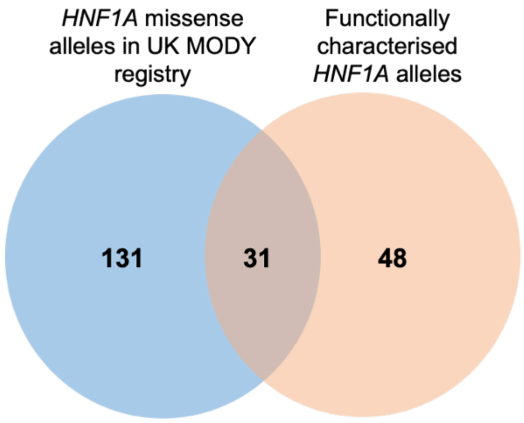

GAD, ICA, IA2, ZnT8 = islet autoantibodies; UCPCR = urinary C-peptide creatinine ratio; OHA = oral hypoglycaemic agent; BMI = body mass index; DM= diabetes mellitus; HbA1c = glycated hemoglobin.

Table S5. Features of *HNF1A* Allele Carriers Documented in Norwegian Registry

Features	Measure
Age at diagnosis	age (years)
Initial Treatment	Diet/OHA/Insulin
Current Treatment	Diet/OHA/Insulin
Sulphonylurea sensitivity	Yes/No
BMI	BMI (kg/m^2)
HbA1c	%
GAD -ve	Yes/No
GAD +ve	Yes/No
ICA +ve	Yes/No
ICA -ve	Yes/No
ZnT8 -ve	Yes/No
ZnT8 +ve	Yes/No
IA2 -ve	Yes/No
IA2 +ve	Yes/No
UCPCR Value	nmol/mmol
C-Peptide	pmol/L
No of generations DM	number of generations with diabetes
Mother DM	Yes/No
Father DM	Yes/No
No of Children DM	number of children with diabetes
Ethnic Origin	ethnicity

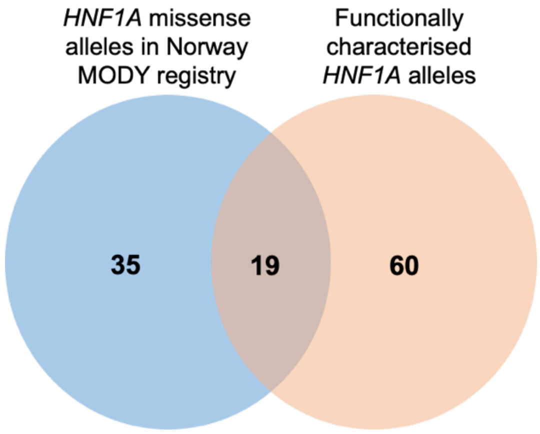

GAD, IA2, ZnT8 = islet autoantibodies; OHA = oral hypoglycemic agent; BMI = body mass index; DM= diabetes mellitus; HbA1c = glycated hemoglobin. Common alleles (AF 3-35%) indicated by a (*).

Table S6. Clinical Features Associated with Functionally-Clinically Discordant *HNF1A* Alleles in UK Registry

(Excel file)

Clinical features and additional information available on each of the listed alleles from the UK MODY diagnostic registry. With the exception of p.R131Q (*n* = 4 documented cases; information displayed as range across the four), details are associated with a single case. Abbreviations: NGS = next generation sequencing; dx = diagnosis; SU = sulfonylurea; FH = family history; BMI = body mass index; HbA1c = glycated hemoglobin.

Table S7. Clinical Features Associated with Functionally-Clinically Discordant *HNF1A* Alleles in Norwegian Registry

(Excel file)

Clinical features and additional information available on each of the listed alleles from the Norway MODY diagnostic registry. Abbreviations: NGS = next generation sequencing; dx = diagnosis; SU = sulfonylurea; FH = family history; BMI = body mass index; HbA1c = glycated hemoglobin
